# Kartogenin‐Conjugated Double‐Network Hydrogel Combined with Stem Cell Transplantation and Tracing for Cartilage Repair

**DOI:** 10.1002/advs.202105571

**Published:** 2022-10-17

**Authors:** You‐Rong Chen, Xin Yan, Fu‐Zhen Yuan, Lin Lin, Shao‐Jie Wang, Jing Ye, Ji‐Ying Zhang, Meng Yang, De‐Cheng Wu, Xing Wang, Jia‐Kuo Yu

**Affiliations:** ^1^ Department of Sports Medicine Beijing Key Laboratory of Sports Injuries Peking University Third Hospital Beijing 100191 China; ^2^ Institute of Sports Medicine Peking University Beijing 100191 China; ^3^ Department of Joint Surgery and Sports Medicine, Zhongshan Hospital Xiamen University Xiamen 361000 China; ^4^ Department of Biomedical Engineering Southern University of Science and Technology Shenzhen 518055 China; ^5^ Beijing National Laboratory for Molecular Sciences State Key Laboratory of Polymer Physics and Chemistry Institute of Chemistry Chinese Academy of Sciences Beijing 100190 China

**Keywords:** cartilage regeneration, cell tracing, double‐network hydrogels, kartogenin, mesenchymal stem cells

## Abstract

The effectiveness of existing tissue‐engineering cartilage (TEC) is known to be hampered by weak integration of biocompatibility, biodegradation, mechanical strength, and microenvironment supplies. The strategy of hydrogel‐based TEC holds considerable promise in circumventing these problems. Herein, a non‐toxic, biodegradable, and mechanically optimized double‐network (DN) hydrogel consisting of polyethylene glycol (PEG) and kartogenin (KGN)‐conjugated chitosan (CHI) is constructed using a simple soaking strategy. This PEG‐CHI‐KGN DN hydrogel possesses favorable architectures, suitable mechanics, remarkable cellular affinity, and sustained KGN release, which can facilitate the cartilage‐specific genes expression and extracellular matrix secretion of peripheral blood‐derived mesenchymal stem cells (PB‐MSCs). Notably, after tracing the transplanted cells by detecting the rabbit sex‐determining region Y‐linked gene sequence, the allogeneic PB‐MSCs are found to survive for even 3 months in the regenerated cartilage. Here, the long‐term release of KGN is able to efficiently and persistently activate multiple genes and signaling pathways to promote the chondrogenesis, chondrocyte differentiation, and survival of PB‐MSCs. Thus, the regenerated tissues exhibit well‐matched histomorphology and biomechanical performance such as native cartilage. Consequently, it is believed this innovative work can expand the choice for developing the next generation of orthopedic implants in the loadbearing region of a living body.

## Graphical abstract

Polyethylene glycol (PEG)‐chitosan (CHI)‐kartogenin (KGN) double‐network (DN) gel combined with peripheral blood‐derived mesenchymal stem cells (PB‐MSCs) is employed to treat knee cartilage defects. Compared with current tissue‐engineering products, it has optimized mechanics, high biosafety, and a simple preparation process. PEG‐CHI‐KGN DN gel promotes the chondrogenic differentiation and survival of PB‐MSCs, ultimately enhancing the regeneration of cartilage defects and providing an innovative clinical treatment strategy from a tissue engineering perspective.

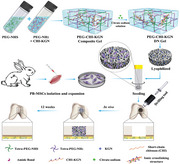

## 1 Introduction

Cartilage injury has increased social and economic burdens as a common joint disease.^[^ ^1^ ^]^ The regeneration of cartilage, an avascular tissue, is often compromised by its lack of innate abilities to mount a sufficient healing response. Current clinical approaches include osteochondral transplantation, bone marrow stimulation, and autologous chondrocyte transplantation, but these strategies are always associated with many inherent drawbacks, such as donor site morbidity, limited availability, potential immunogenic rejection, and disease transmission.^[^ ^2^ ^]^ Fortunately, tissue engineering technology, which relies on the optimized regulation of biological scaffolds, growth factors, and seed cells, provides novel therapeutic modalities for cartilage repair and tissue reconstruction.^[^ ^3^ ^]^ Given the comprehensive sources of biomaterials and seed cells, which are conducive to large‐scale cultivation in vitro, tissue‐engineering cartilage owns advantageous capacities to repair large tissue defects and avoid donor complications and two‐stage invasive surgical procedures.^[^ ^4^ ^]^

Hydrogels are emerging as a promising biological scaffold due to their hydrated 3D architectures, better biocompatibility, biodegradability, bioactivity, and abundance from diverse sources.^[^ ^5^ ^]^ However, inherently low mechanical strength greatly precludes their applications, especially in load‐bearing tissue substitutes.^[^ ^6^ ^]^ Among a wide array of hydrogels for tissue engineering, double‐network (DN) hydrogels, consisting of two interpenetrating networks with strong contrasting physical properties, possess better mechanical properties that can provide essential mechanical support and biomechanical cues to maintain the phenotype of cartilage‐forming cells for promoting cartilage repair.^[^ ^7^ ^]^ Nevertheless, in most cases, biocompatible DN gels alone are not chondrogenic in nature. To address this shortcoming, stem cells, especially mesenchymal stem cells (MSCs), and growth factors are incorporated using various strategies to promote tissue regeneration.^[^ ^8^ ^]^ Compared to frequently‐used bone marrow‐derived MSCs (BM‐MSCs), peripheral blood‐derived MSCs (PB‐MSCs) have distinct biological advantages of being more minimally invasive, fewer complications, repeatable sampling, and similar cartilage differentiation tendency, which may be competitive seed cells for the effective cartilage repair and tissue regeneration.^[^ ^9^ ^]^ Although the hydrogel scaffolds can incorporate multiple growth factors/peptides to generate heterogeneous tissue constructs and achieve multiple functions, several potential concerns are associated with the growth factor approach, including short half‐life, protein instability, undesired dose‐related side effects, and higher costs.^[^ ^10^ ^]^ Compared to the short half‐life of biological protein growth factors, kartogenin (KGN) as a nonprotein small molecule chondrogenesis inducing agent, was first reported by Johnson et al. in 2012.^[^ ^11^ ^]^ They screened 22 000 structurally diverse and heterocyclic drug‐like small molecules and found that KGN was capable of significantly promoting chondrocyte differentiation of human MSCs in a dose‐dependent manner without any toxicity. Most importantly, KGN can be stored and transported at room temperature as a very stable small molecule. The obvious superiorities compared to protein growth factors make KGN a potential chondrogenesis promoter in cartilage tissue engineering.^[^ ^11^, ^12^ ^]^ Therefore, developing biodegradable and mechanically optimized DN gel scaffolds to guide the controlled release of bioactive molecules and promote the ingrowth of cartilage regeneration is highly desired. However, it usually involves complicated preparation steps, toxic initiators, and a tedious modulation process.^[^ ^13^ ^]^

**Scheme 1:**
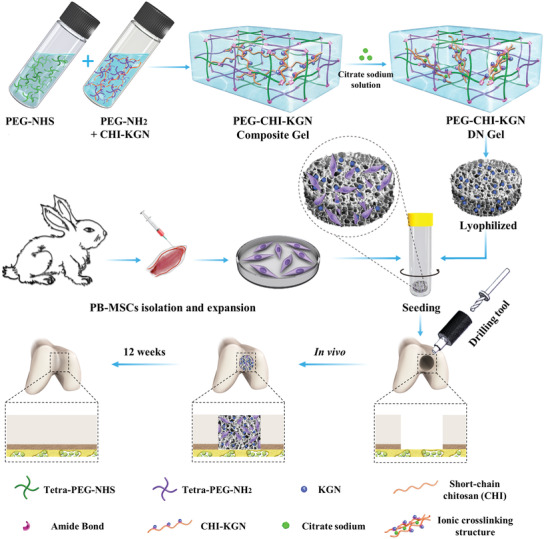
Schematic of PB‐MSCs‐loaded PEG‐CHI‐KGN DN gel for cartilage repair.

Herein, to address the above challenges, we first integrated KGN‐conjugated chitosan (CHI) into a covalent tetra‐armed polyethylene glycol (PEG) network via hydrogen bonding to construct a PEG‐CHI‐KGN composite hydrogel, and then, applied a simple soaking strategy to convert the composite hydrogel into a mechanically optimized PEG‐CHI‐KGN DN gel via the formation of CHI ionic networks by virtue of coordination interaction and secondary interaction in Na_3_Cit solutions.^[^ ^7a^ ^]^ The PEG‐CHI‐KGN DN gel displayed suitable mechanical strength and favorable cell attachment and growth. On account of the suitable pore structure, swelling ratio and degradation time of DN gel, KGN molecules were sustainedly released from the hydrogel scaffolds over 6 weeks, thus effectively promoting the chondrogenic differentiation of PB‐MSCs. After in vivo implantation for 12 weeks, this non‐toxic, bioactive, mechanically, and functionally optimized PEG‐CHI‐KGN DN gel combined with PB‐MSCs could significantly promote cartilage repair and tissue regeneration (**Scheme** **1**). The regenerated tissues were close to natural cartilage based on the histological tests, specific markers analysis, and biomechanical tests. Of note, employing the reliable surveillance technique of sex‐determining region Y‐linked gene sequence (SRY), this is the first time we clarified that allogeneic PB‐MSCs could survive in cartilage defects for a long time in vivo to facilitate cartilage regeneration. Given the higher proportion of allogeneic PB‐MSCs in regenerated cartilage of the PEG‐CHI‐KGN group, we further elucidated the potential molecular mechanism of KGN in cartilage repair by RNA sequencing. The PEG‐CHI‐KGN DN gel has optimized mechanics, reasonable microstructure, high biosafety, chondrogenic microenvironment, and a simple preparation process, which has great clinical application and translational potential. This study provided a potential and innovative clinical treatment strategy on tissue engineering therapy for cartilage defects and deep insight into regenerative medicine.

## 2 Results and Discussion

### 2.1 Preparation and Characterization of PEG‐CHI and PEG‐CHI‐KGN Hydrogels

Scheme 1 shows the simple preparation process of hybrid PEG‐CHI‐KGN DN hydrogels by post‐crosslinking PEG‐CHI‐KGN composite hydrogel using the soaking strategy in our previous works.^[^ ^14^ ^]^ Briefly, highly ductile PEG‐CHI and PEG‐CHI‐KGN composite hydrogels were prepared by simply mixing the four‐arm‐PEG‐NH_2_, CHI or CHI‐KGN, and four‐arm‐PEG‐NHS solutions (Figure S1A, Supporting Information). The gelation time of PEG‐CHI‐KGN was obviously shorter than that of PEG‐CHI composite gel (Figure S1B, Supporting Information), which may ascribe to the reduced amine groups that could impair the hydrogen interactions among the CHI chains and water molecules and decrease the solution viscosity. Then, the composite gels were immersed in saturated sodium citrate solution overnight to produce the ion crosslinking physical network of CHI via coordination and secondary interactions and obtain the ionic‐covalent PEG‐CHI DN gel and PEG‐CHI‐KGN DN gels.

**Figure 1:**
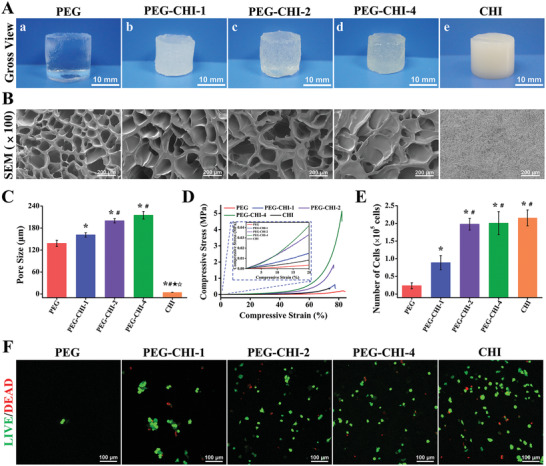
A) Gross view of PEG hydrogel, PEG‐CHI DN gels, and CHI hydrogel. B) Scanning electron micrographs of hydrogels to observe the microstructures. C) The average pore sizes of five hydrogels (*n* = 3). D) Unconfined compressive mechanical tests of five hydrogels. E) The number of PB‐MSCs on hydrogels after seeding for 3 days (*n* = 5). F) Live/Dead staining of PB‐MSCs on various hydrogels. (*P < 0.05 versus PEG, **^#^** *P <* 0.05 versus PEG‐CHI‐1, ^★^ *P <* 0.05 versus PEG‐CHI‐2, and ^☆^ *P <* 0.05 versus PEG‐CHI‐4).

As the CHI contents and KGN grafting ratio could directly affect the structure and property of DN gels, we first prepared three composite hydrogels, PEG‐CHI‐1, PEG‐CHI‐2, and PEG‐CHI‐4 with various CHI contents (1 wt%, 2 wt%, and 4 wt%) and further obtained corresponding PEG‐CHI DN Gels by post‐crosslinking PEG‐CHI composite hydrogel using the soaking strategy. In contrast, the chemically crosslinked tetra‐PEG hydrogels and physically crosslinked CHI ionic hydrogels were also obtained as controls in **Figure** **1**A. Along with the increase of CHI contents, the pore size and mechanical strength (compression elastic modulus and compression strain) of PEG‐CHI DN gels were enhanced in Figure 1B–D. However, the poor pore interconnection of PEG‐CHI‐4 constrained the entry capacity of cells into a hydrogel and cell proliferation (Figure 1E,F; Figure S2A,B, Supporting Information). We further verified the effect of the microstructure of different hydrogels on the chondrogenic differentiation of PB‐MSCs. The results showed that type II collagen (COL‐2) of PB‐MSCs‐PEG‐CHI‐2 DN hydrogel complex was closer to native chondrocytes after chondrogenic differentiation (Figure S2C,D, Supporting Information). Taken together, we chose PEG‐CHI‐2 DN gels for further investigation of structures and properties.

**Figure 2:**
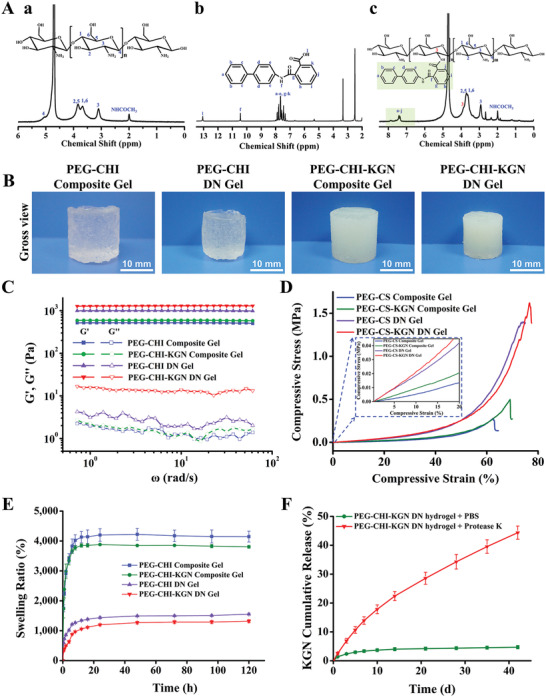
A) Typical ^1^H NMR spectra of a) CHI, b) KGN, and c) CHI‐KGN conjugate. B) Gross view of PEG‐CHI and PEG‐CHI‐KGN composite and DN gels. C) Unconfined compressive mechanical tests of four hydrogels. D) Rheological measurements of oscillatory frequency sweep experiments were used to assess the shear modulus of hydrogels. E) Swelling kinetics of hydrogels in PBS at each time point (*n* = 3). F) Cumulative release of KGN from PEG‐CHI‐KGN DN gels at each time point (*n* = 5).

KGN, as a nonprotein small molecule chondrogenesis inducing agent, played an essential role in constructing functional hydrogel for cartilage regeneration. CHI‐KGN conjugate was feasibly prepared with a grafting ratio of 92.97 ± 1.15% by the high‐effective amidation reaction between the EDC/NHS‐activated ester of KGN and amine group of CHI, as testified by the ^1^H NMR spectra in **Figure** **2**A. The major signals of benzene groups at *δ* = 7.3–7.9 ppm for KGN and prominent resonance peaks at *δ* = 1.9 and 2.7 ppm corresponding to the methyl (—CH_3_) and methylene proton at the C3 position of CHI confirmed the successful preparation of CHI‐KGN conjugate. According to the above‐mentioned post‐crosslinking strategy, the PEG‐CHI‐KGN composite and ionic‐covalent DN gels (CHI/CHI‐KGN, 2 wt%) were prepared with lower transparency than PEG‐CHI hydrogels (Figure 2B). As shown in Figure S3A‐C, Supporting Information, the pore sizes and porosities of PEG‐CHI DN gels (199.83 µm, 77.44 ± 3.84%) and PEG‐CHI‐KGN DN gels (202.69 µm, 76.61 ± 3.53%) could satisfy the pore requirement of engineering scaffolds (≈200 µm) for the advantageous chondrogenic differentiation of MSCs.^[^ ^15^ ^]^ Compared to the composite gels, smaller pore sizes and porosities of DN gels were attributed to the formation of ionic network and volume shrinkage during the immersion in saturated salt solutions, which had a crucial impact on the physical properties of hydrogels.

As shown in Figure 2C, all samples possessed a single plateau region in their dynamic moduli. The storage modulus (*G*′) values had a substantial elastic response and were always larger than the dissipative modulus (*G*′′) values over the entire range of frequencies. Higher storage modulus *G*′ of PEG‐CHI and PEG‐CHI‐KGN DN gels indicated the formation of well‐ordered microstructures and CHI ionic network by the strong *N*‐glucosamine‐Cit^3−^ tridentate interaction.^[^ ^14^ ^]^ The tiny *G*′ difference between the PEG‐CHI and PEG‐CHI‐KGN DN gels may be ascribed to the rigid backbone of KGN components. In addition, compressive curves quantitatively evaluated the enhancement of mechanical strength for DN gels. The combination of tetra‐PEG and CHI overcame the shortcomings of single tetra‐PEG in softness, and CHI hydrogels in brittleness. The PEG‐CHI‐KGN DN gels have the highest compressive strength (1.62 MPa), compressive strain (>75%), and compressive elastic modulus (254 kPa) among these four hydrogels (Figure 2D; Figures S3D and S4, Supporting Information), ensuring its stability even under external forces.

To reveal the energy dissipation capacity of the PEG‐CHI, PEG‐CHI‐KGN composite gels, and their DN gels, we investigated the stress–strain curves of hydrogels using cyclic loading experiments. Figure S5, Supporting Information, shows that the DN gels had a larger hysteresis loop than the composite hydrogels in the first cycle, confirming the more‐efficient energy dissipation of DN gels. Then, the second loading–unloading curves were immediately conducted, and the hysteresis loop of DN gels became smaller, indicating the softening occurrence. In contrast, the composite hydrogels showed overlapped hysteresis loops for the two cycles, demonstrating a typical rubber elastic behavior. We deduced that when the DN gels were compressed, the loose CHI/CHI‐KGN network could rupture into small clusters to dissipate the energy, contributing to the larger hysteresis loop.^[^ ^16^ ^]^ The cyclic loading experiment results robustly illustrated that incorporating CHI/CHI‐KGN into tetra‐PEG hydrogels indeed improved the mechanical properties of the PEG‐CHI and PEG‐CHI‐KGN DN gels, which were critical as an implantable tissue engineering scaffold for load bearing.

Figure 2E displays that all hydrogels could reach the swelling equilibrium after incubating at PBS for 24 h. Compared with PEG‐CHI and PEG‐CHI‐KGN composite gels, PEG‐CHI and PEG‐CHI‐KGN DN gels showed a lower swelling ratio (1553% and 1316%) and maintained their stable swelling behaviors even after 120 h in PBS, indicating the more stable structures of DN gels. The same interpretation was that the newly formed physical skeleton and tridentate coordination interactions contributed to a much denser structure of hydrogel, which restricted the diffusion of water molecules into the network to a certain degree.

PBS solution containing protease K was used as the medium for hydrogel degradation to simulate the degradation process of hydrogel in vivo. As shown in Figure S6, Supporting Information, in PBS solution without protease K, there was only 15.67% and 14.03% mass loss of PEG‐CHI and PEG‐CHI‐KGN DN hydrogels after 6 weeks, respectively. In the protease K treatment groups, the mass loss of PEG‐CHI and PEG‐CHI‐KGN DN gels was as high as 60.48% and 55.89% after 6 weeks. It was related to the fact that protease K could effectively promote the degradation of the amide and ester bonds, indicating the applicable degradation rate of PEG‐CHI‐KGN DN gels in vivo.^[^ ^17^ ^]^ Furthermore, we evaluated the KGN release profile from PEG‐CHI‐KGN DN gels in PBS solution with or without protease K using UV–vis spectrophotometry. Figure 2F reveals that the PEG‐CHI‐KGN DN gels got a well‐controlled release of KGN in PBS solution with protease K and 44.36% of KGN release from the DN gels after 6 weeks could completely satisfy its bio‐effective concentration requirement in vivo (100 nm–100 µm).^[^ ^12^, ^18^ ^]^

### 2.2 In Vitro Biological Characterization of PB‐MSCs‐Laden PEG‐CHI KGN DN Gels

PB‐MSCs were isolated by the method of density gradient centrifugation and cell adhesion.^[^ ^19^ ^]^ Several PB‐MSCs clusters began to appear after the culture of initial 12–14 days and were then transformed into typical spindle morphology. After incubation for 21 days, these primary cells achieved ≈80–90% confluence. PB‐MSCs exhibited a relative homogeneity at passage 3 (Figure S7a, Supporting Information). Multilineage differentiation potential of PB‐MSCs was identified by the tri‐lineage differentiations method. Alizarin red staining was used to detect the deposition of calcium nodules to confirm the osteogenic differentiation after 14 days (Figure S7b, Supporting Information). Oil red O staining showed the accumulation of lipid vacuoles in cells at 21 days, which suggested the adipogenic differentiation of PB‐MSCs (Figure S7c, Supporting Information). A spherical pellet was formed under the condition of micromass culture at 3 days. Alcian blue staining detected cartilage‐specific aggregating proteoglycans and determined the chondrogenic differentiation potential of PB‐MSCs after incubation for 21 days (Figure S7d, Supporting Information). An increasing number of studies reported that the PB was a potential alternative source of MSCs for the proliferation and chondrogenic differentiation as BM‐MSCs both in vitro and in vivo.^[^ ^20^ ^]^

To detect the cytotoxicity of hydrogels, PB‐MSCs were cultured in a medium containing extracting liquid of PEG‐CHI and PEG‐CHI‐KGN DN gels for 24 and 48 h. CCK‐8 assay in Figure S8A, Supporting Information, demonstrates that both PEG‐CHI and PEG‐CHI‐KGN DN gels possessed good biocompatibility with cell viability of over 97%. Figure S8B, Supporting Information, reveals the proliferation of PB‐MSCs on PEG‐CHI and PEG‐CHI‐KGN DN gels during the first week. There was no statistical difference between the two groups on days 1 and 4 (*n* = 5, *P >* 0.05). However, PB‐MSCs cultured on PEG‐CHI‐KGN DN gel showed slower proliferation rate than that on PEG‐CHI DN gel on day 7 (*n* = 5, *P < 0.05). After 7 days of culture in cell medium, Live/Dead staining showed that the majority of seeded cells survived in the scaffolds with few dead cells, further confirming the low toxicity of these four biocompatible hydrogels. The number of cells in the PEG‐CHI DN gel group seemed slightly more than that in the PEG‐CHI‐KGN DN gel group (Figure S8C, Supporting Information). It has been reported that the proliferation rate of chondrocytes is slower than that of MSCs.^[^ ^21^ ^]^ Therefore, we speculated that stronger chondrogenic differentiation properties of PEG‐CHI‐KGN DN gel could result in a slower proliferation of PB‐MSCs.

**Figure 3:**
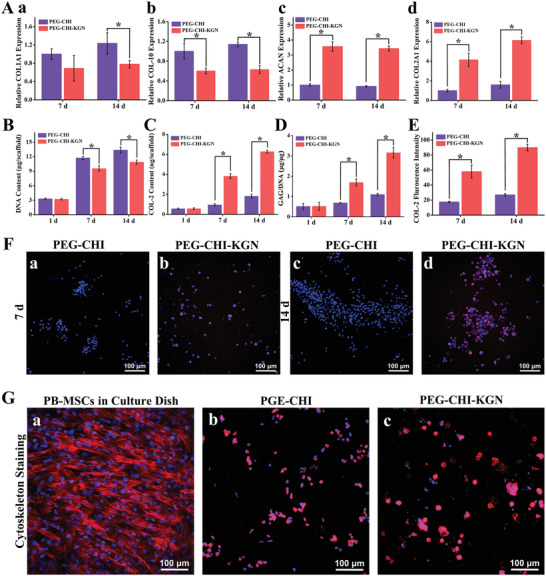
A) The expression of fibrotic related genes COL1A1, hypertrophic gene COL‐10, and cartilage‐specific genes COL2A1 and ACAN (*n* = 3, *P < 0.05). The contents of B) DNA, C) COL‐2, and D) GAG in PEG‐CHI and PEG‐CHI‐KGN DN gels (*n* = 3, *P < 0.05). E) Immunofluorescent staining visualized the production of COL‐2 in the PB‐MSCs‐hydrogel composites after 14 days of in vitro culture (Blue: DAPI; Red: COL‐2). F) Fluorescence intensity of COL‐2 in the PB‐MSCs‐hydrogel composites (*n* = 3, *P < 0.05). G) Cytoskeleton staining revealing morphology of PB‐MSCs in a culture dish (2D) and hydrogels (3D) (Blue: DAPI; Red: Phalloidin).

We analyzed the expression of fibrotic‐related genes COL1A1, hypertrophic gene COL‐10, and cartilage‐specific genes COL2A1 and aggrecan (ACAN) (**Figure** **3**A). Compared to the PEG‐CHI group, the PEG‐CHI‐KGN group exhibited lower expression of COL1A1 and COL‐10 and higher expression of COL2A1 and ACAN on days 7 and 14, which suggested that PEG‐CHI‐KGN could promote the expression of cartilage‐specific genes and effectively inhibit cell aging. Figure 3B shows that the DNA contents continued to increase over 2 weeks of culture, and the average DNA contents in the PEG‐CHI group were higher than the PEG‐CHI‐KGN group on days 7 and 14. The contents of COL‐2 and glycosaminoglycan (GAG) in PEG‐CHI and PEG‐CHI‐KGN DN gels were quantitatively evaluated via the ELISA analysis and 1, 9‐dimethylmethylene blue assay. Figure 3C,D shows that the contents of COL‐2 and GAG in the PEG‐CHI‐KGN DN gel group increased continuously over time, and reached its maximum amount at day 14, which was higher than the PEG‐CHI group.

Immunofluorescent staining demonstrated the production of COL‐2 protein in cell–hydrogel composites. After culturing for 7 and 14 days, COL‐2 expression in PEG‐CHI‐KGN group was stronger than in the PEG‐CHI group, along with the higher fluorescence intensity of COL‐2 (Figure 3E,F), which was consistent with the ELISA analysis. It was attributed to the long‐term release of KGN that was more conducive to the expression of cartilage‐specific genes, the secretion of extracellular matrix in PB‐MSCs, and chondrogenesis of MSCs. KGN had been testified to have abilities to regulate the core‐binding factor *β* subunit (CBF*β*)‐RUNX1 transcription program to induce chondrogenesis by binding filamin A, disrupting its interaction with the transcription factor CBF*β*.^[^ ^11^ ^]^ Cytoskeleton staining was used to observe the morphology of PB‐MSCs in DN gels after culturing for 14 days. PB‐MSCs were shifted from a spindle‐like morphology in the 2D culture dish (Figure 3G‐a) to a rounded chondrocyte‐like shape in hydrogels, especially in the PEG‐CHI‐KGN group (Figure 3G‐b,c). This fully demonstrated the 3D structure of PEG‐CHI and PEG‐CHI‐KGN DN gel scaffolds was beneficial to promoting PB‐MSCs to maintain chondrocyte‐like morphology.

### 2.3 Cartilage Repair Efficacy of the DN Gels In Vivo

**Figure 4:**
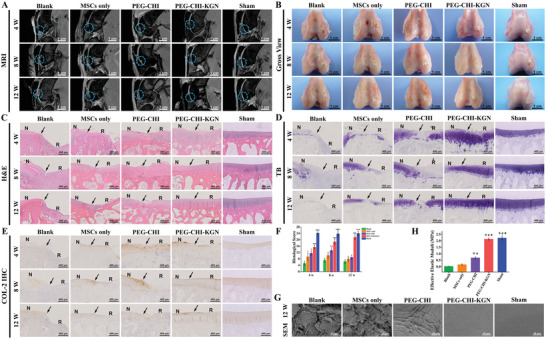
A) MRI detection of specimens after the operation of cartilage repair. B) Macroscopic observation of the repaired cartilage defects at various time points. C) H&E staining of repaired cartilage at 4, 8, and 12 weeks (N: normal cartilage; R: repair cartilage; the arrows indicate the margins of the normal cartilage and repaired cartilage). D) Toluidine blue staining of repaired cartilage at 4, 8, and 12 weeks. E) Immunohistological staining for COL‐2 at 4, 8, and 12 weeks. F) The histological score for repaired cartilage during in vivo implantation (*n* = 5). G) SEM images of the surface of the repaired sites at 12 weeks after implantation. H) Biomechanical properties of repaired cartilage in different groups (*n* = 3), (*P < 0.05 versus Blank group, **^#^** *P <* 0.05 versus MSCs only group, ^★^ *P < 0.05* versus PEG‐CHI group, and ^☆^ *P <* 0.05 versus PEG‐CHI‐KGN group).

For the in vivo experiments, the cartilage defect was carried on the trochlear groove by the modified corneal trephine. The PB‐MSCs, PB‐MSCs‐PEG‐CHI DN gel complexes, or PB‐MSCs‐PEG‐CHI‐KGN DN gel complexes were transplanted to repair the rabbit cartilage defects (MSCs only group, PEG‐CHI group, and PEG‐CHI‐KGN group). In the Blank group, the cartilage defects were left empty and served as the control. In the Sham group, the knee incision was immediately sutured without making cartilage defects as a control. A total of five rabbits died of diarrhea during the feeding process (one in Blank group, two in MSCs only group, one in PEG‐CHI group, and one in PEG‐CHI‐KGN group). We then supplemented rabbits in the corresponding groups. After the repair operation, the specimens were examined using MRI at different time points (**Figure** **4**A). The five groups were compared by observing the filling of regenerated tissue at defects, the fusion with surrounding cartilage, subchondral bone signal, intra‐articular inflammation, and surrounding cartilage degeneration.

High resolution MRI showed that there was an obvious defect area in the Blank group at 4 weeks, which was larger than that in the MSCs only group. In the PEG‐CHI group, although the defect area was more filling, it did not fuse well with the cartilage around the defect, and an obvious abnormal high signal shadow was observed in subchondral bone. In the PEG‐CHI‐KGN group, the cartilage defect area was relatively full and fused well with the surrounding cartilage, but the thickness was thinner than that of the Sham group, and there was a slight abnormal high signal shadow in subchondral bone. At 8 weeks, the defect area in the Blank group was partially filled with disorganized regenerated tissue, exhibiting an obvious abnormal high signal shadow in the subchondral bone. In MSCs only group, the defect area was filled with regenerated tissue. Although the signal of regenerated tissue was similar to that of surrounding cartilage, the surface was obviously uneven and poorly fused with surrounding cartilage, and the flake high signal shadows were observed in subchondral bone. In PEG‐CHI group, the cartilage defect area was basically filled with regenerated tissue, but the fusion with the surrounding cartilage was poor, and the abnormal high signal shadow of subchondral bone still existed. In PEG‐CHI‐KGN group, the defect area was basically filled with no abnormal signal shadow in subchondral bone which demonstrated its effective fusion with the surrounding tissues though the thickness was still thinner than that of Sham group. At 12 weeks, the defect area in the Blank group was filled with structurally disordered regenerated tissue, along with the irregular surface, serious inflammation in the articular cavity, and uneven surrounding cartilage signal. In MSCs only group, the defect area was filled with cartilage like signal tissue, but the thickness was significantly thinner than that of Sham group, indicating the unqualified fusion with surrounding cartilage, which was similar with PEG‐CHI group. In contrast, the regenerated cartilage in PEG‐CHI‐KGN group with uniform signal and smooth surface was similar to Sham group.

Gross observations and International Cartilage Repair Society (ICRS) score (Table S3, Supporting Information) were performed for the macroscopic evaluation of cartilage repair. Gross observations basically echoed the MRI results which showed poor cartilage regeneration in the Blank group. The regenerated cartilages in the MSCs only group and the PEG‐CHI group were better than that of the Blank group, but overall it was worse than the PEG‐CHI‐KGN and Sham group. The cartilage in the PEG‐CHI‐KGN group showed much better filling of regenerative tissue, smoother surface, and less degeneration of surrounding cartilage (Figure 4B). The ICRS scores of Blank group, MSCs only group, and PEG‐CHI group were first increased and then decreased, and the scores were the highest at 8 weeks. The score of PEG‐CHI‐KGN group continued to rise over time and was the highest at 12 weeks. The score of Sham group remained stable at different time points. At 12 weeks, the repair effects of PEG‐CHI group and PEG‐CHI‐KGN group were better than the Blank group, and PEG‐CHI‐KGN group close to Sham group. There was no significant difference in the scores between MSCs only group and PEG‐CHI group (Figure S9A, Supporting Information). These results showed that functionalized PEG‐CHI‐KGN DN gel combined with allogeneic PB‐MSCs could effectively repair the cartilage defects without detaching during joint movement, thereby promoting the regeneration and remodeling of articular cartilage defects.

HE staining, toluidine blue (TB) staining, and immunohistochemistry (IHC) for COL‐2 were also used to assess the quality of tissue repair. HE staining showed that the cartilage defect persisted in the Blank group, and the repaired tissue was fibrous tissue with disordered structure, accompanied by inflammatory cell infiltration, poor repair of subchondral bone, and obvious degeneration of the surrounding cartilage. The regenerated cartilage and surrounding cartilage in the MSCs only group showed moderate degeneration, with large cracks on the surface, and incomplete repair of subchondral bone. The cartilage defect in the PEG‐CHI group was filled with regenerated tissue, but the surface structure was disordered. In contrast, the PEG‐CHI‐KGN group showed continuous cartilage repair and regeneration. At 12 weeks, the thickness and structure of the regenerated cartilage in the PEG‐CHI‐KGN group were similar to Sham group, and the surface was smooth (Figure 4C). TB staining and semi‐quantitative analysis showed that the chondroid ECM in the repaired area of the Blank group and MSCs only group was enhanced at the initial stage and then weakened over time, presenting mild staining or even staining loss, and the degeneration of cartilage around the defect gradually occurred. In PEG‐CHI group, the thickness of the regenerated tissue at the repaired site first increased and then decreased, and the staining of surrounding cartilage was weakened at 12 weeks, suggesting the degeneration process and evolution. The staining and thickness of repaired tissues in PEG‐CHI‐KGN group increased gradually with time. At 12 weeks, the ECM staining of the regenerated cartilage in the PEG‐CHI‐KGN group was uniform, comparable to that of Sham group (Figure 4D; Figure S9B, Supporting Information). IHC staining and semi‐quantitative analysis were basically consistent with toluidine blue staining, indicating superior cartilage repair quality for the PEG‐CHI‐KGN DN gel without hypertrophic cartilage remodeling compared to the Blank, MSCs only, and PEG‐CHI groups (Figure 4E **;** Figure S9C, Supporting Information). Wakitani histological score was performed to measure the degree and quality of cartilage repair (Table S4, Supporting Information). PEG‐CHI‐KGN group had the highest histological score at 12 weeks, significantly higher than those of the Blank, MSCs only, and PEG‐CHI groups at any time point (*n* = 5, *P* < 0.05, Figure 4F). To evaluate the presence of inflammatory response, synovial fluid was collected for analysis of interleukin‐1*β* (IL‐1*β*) and tumor necrosis factor‐*α* (TNF‐*α*). The levels of inflammatory factors in the PEG‐CHI‐KGN and Sham groups started to decline and were maintained at a relatively low level after 4 weeks during the repair process. The quantification of inflammatory cytokines in the PEG‐CHI‐KGN and Sham groups reached the lowest at 12 weeks (Figure S10A,B, Supporting Information), indicating its satisfactory tissue regeneration.

Besides, we also performed the microscopic observation and biomechanical test on the repaired defect areas by nanoindentation (Figure 4G,H). At 12 weeks after operation, the obvious deep cracks and uneven surface were seen in the Blank and MSCs only groups. In the PEG‐CHI group, shallower cracks were displayed on the surface of regenerated cartilage and some fiber‐like tissue irregularly filled the defects. In contrast, the PEG‐CHI‐KGN group exhibited a smooth and homogeneous surface that was highly similar to the microstructure of the Sham group.^[^ ^22^ ^]^ More importantly, the Young's modulus of regenerated cartilage in PEG‐CHI‐KGN group (2.14 ± 0.85 MPa) was significantly higher than that in the Blank, MSCs only, and PEG‐CHI groups (*n* = 3, *P* < 0.05), which was approximate to the Sham group (2.23 ± 0.16 MPa, *n* = 3, *P* > 0.05). Overall, these results mentioned above adequately revealed that the PEG‐CHI‐KGN DN gel could promote the well‐organized cartilage repair with explicit morphological structure and favorable biomechanical strength as normal native cartilage.

### 2.4 Fluorescent In Situ Hybridization (FISH) Detection of Allogeneic PB‐MSCs

Tracking the proliferation, differentiation, and survival of MSCs in the implantation area was one of the most critical issues in stem cell transplantation research.^[^ ^23^ ^]^ In our study, the SRY was chosen as a marker to monitor the transplanted allogeneic stem cells.^[^ ^24^ ^]^ FISH showed the positive intra‐nuclear signals in the knee articular cartilage of male rabbits (positive control, Figure S11A‐a,c,e, Supporting Information) and negative intra‐nuclear signals in the knee articular cartilage of female rabbits (negative control, Figure S11A‐b,d,f, Supporting Information), which could confirm the specificity of chosen mRNA probe. FISH was performed on regenerated cartilage to detect engrafted allogenic PB‐MSCs after surgery for 4, 8, and 12 weeks.

**Figure 5:**
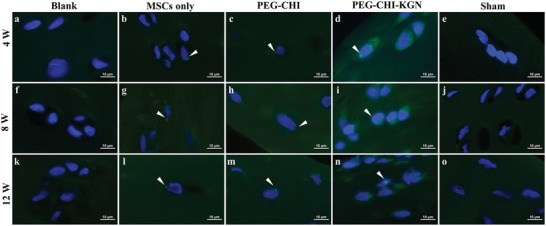
Fluorescent in situ hybridization (FISH) performed on PB‐MSCs engraftments at 4, 8, and 12 weeks after surgery. No intranuclear positive signal was found in the Blank group and Sham group. A small number of intranuclear signals was positive at each time point in MSCs group, PEG‐CHI group, and PEG‐CHI‐KGN group (Blue: DAPI, Green: FISH).

There was no intranuclear positive signal in the Blank and Sham groups. A small number of intranuclear signals were positive at each time point in the MSCs only group, the PEG‐CHI group, and the PEG‐CHI‐KGN group, but the positive signal and proportion of stem cells in regenerated tissues decreased over time (**Figure** **5** **;** Figure S11B, Supporting Information). The proportion of stem cells in regenerated tissues of the MSCs only group and in the PEG‐CHI group showed no significant difference but was significantly lower than that in PEG‐CHI‐KGN group (*n* = 3, *P* < 0.05) (Figure S11C, Supporting Information). Survival of transplanted stem cells was limited by apoptosis, hypoxia tolerance, and the immune and inflammatory response.^[^ ^25^ ^]^ Our analysis suggested that PEG‐CHI‐KGN DN hydrogel could sustainedly release the KGN, promote PB‐MSCs chondrogenic differentiation and repair cartilage defects, and allow these transplanted PB‐MSCs more resistance to hypoxia and lower inflammatory reaction in the joint cavity. Although the proportion of allogeneic PB‐MSCs in regenerated cartilage was lower than that of host cells, this finding suggests they could survive in cartilage defects for a long time and contribute to cartilage regeneration, especially in the early stages of cartilage repair. For eventual clinical applications, the use of autologous or bank‐matched stem cells would be expected to reduce immune response further and prolong cell survival.^[^ ^26^ ^]^ This SRY detecting technique was demonstrated to be effective and reliable, which could avoid the attenuation of markers, high false positives, cytotoxicity, and tumorigenicity caused by other tracing methods such as a magnetic probe, fluorescent dye, and nanoparticles.^[^ ^27^ ^]^ However, the limitation of this approach is that it only applied to male cells in female hosts.^[^ ^28^ ^]^

### 2.5 The Molecular Mechanisms of KGN to Enhance Cartilage Regeneration

**Figure 6:**
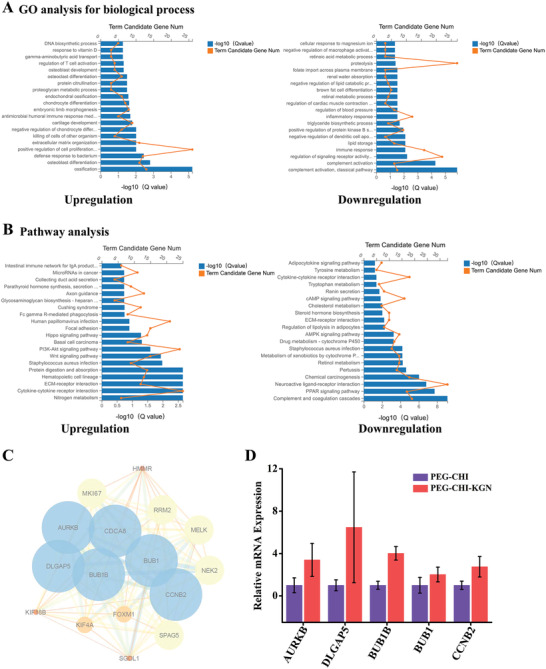
A) GO analysis for differentially expressed genes of cartilage transcriptome in regenerated cartilage of PEG‐CHI and PEG‐CHI‐KGN groups at 12 weeks after surgery. B) Pathway analysis for differentially expressed genes from the rabbit regenerated cartilage transcriptome. C) The most significant clustered module of differentially expressed genes was identified from the PPI network utilizing MCODE in Cytoscape. The more size nodes shown, the more degree nodes obtained. Interactions were color coded according to the combined scores, with darker edges corresponding to higher scores. D) RT‐PCR validation for essential genes in the most significant cluster in RNA‐Seq; data were expressed as mean ±SD (*n* = 3, *P* < 0.05).

To explore the potential molecular mechanisms of KGN to enhance cartilage formation, we performed RNA sequencing (RNA‐seq) to analyze gene expression profiles of regenerated cartilage of PEG‐CHI and PEG‐CHI‐KGN DN gels. The results showed 1174 gene expression differences between the two groups (Figure S12, Supporting Information). Gene ontology (GO) analysis indicated that DNA biosynthetic process, chondrocyte differentiation, cartilage development, and positive regulation of cell proliferation were upregulated, while terms including proteolysis, and inflammatory and immune response were downregulated (**Figure** **6**A). KEGG pathway analysis revealed that glycosaminoglycan biosynthesis–heparan sulfate/heparin, focal adhesion, ECM‐receptor interaction, and nitrogen metabolism were significantly upregulated, which regulated the synthesis of cartilage extracellular matrix, cell adhesion, and chondrocyte proliferation and differentiation.^[^ ^29^ ^]^ Meanwhile, adipocytokine signaling pathway, complement and coagulation cascades, and PPAR signaling pathway were downregulated (Figure 6B), reducing the local inflammation and immune response and inhibiting the adipogenic differentiation.^[^ ^30^ ^]^

Molecular complex detection (MCODE) was performed to screen the modules of a large PPI network. The most significant clustered module of differentially expressed genes was identified; this module contained 16 genes and 111 interaction protein pairs (Figure 6C), mainly related to DNA biosynthetic process, cell cycle, and cell proliferation and differentiation. According to the most significant cluster module of differentially expressed genes, 6 essential genes (AURKB, DLGAP5, CDCA8, BUB1B, BUB1, and CCNB2) were selected for the quantitative real‐time PCR (RT‐PCR) validation. Among them, the expression of five upregulated genes (AURKB, DLGAP5, BUB1B, BUB1, and CCNB2) in the PEG‐CHI‐KGN group was higher than that in the PEG‐CHI group, which was consistent with the RNA‐seq results (Figure 6D). These differentially expressed genes might be involved in regulating chondrocyte proliferation and fate.^[^ ^31^ ^]^

We further cultured PB‐MSCs in medium with or without KGN for 2 weeks and detected the protein secretion of AURKB, DLGAP5, BUB1B, BUB1, CCNB2, and CDCA8 by immunofluorescence staining (IF). The results showed that the protein expression in the KGN group was higher than that in the control group (*n* = 3, *P < 0.05) (Figure S13, Supporting Information). At the same time, the protein expression of regenerated cartilage in PEG‐CHI group and PEG‐CHI‐KGN group at 12 weeks was detected by IHC staining. The results of semi‐quantitative analysis showed that the protein expression of PEG‐CHI‐KGN group was higher than that of PEG‐CHI group (*n* = 3, *P* < 0.05) (Figure S14, Supporting Information). The above results further validate the potential molecular mechanisms of KGN to enhance cartilage formation and provide an insight into the molecular mechanisms of KGN in cartilage repair and tissue regeneration.

## 3 Conclusion

In summary, a novel non‐toxic PEG‐CHI‐KGN DN gel with attractive mechanical strength and biological activity was successfully prepared by in situ gelation and ion immersion strategy. These DN gels possessed highly interconnected porous microstructure, sizes and porosities, and appropriate swelling ratio and biodegradability, which could favor the attachment, ingrowth, and proliferation of living cells. In addition, the sustained release of KGN could effectively promote the chondrogenic differentiation of PB‐MSCs and secretion of cartilage ECM, thus facilitating the filling of the defects and the generation of cartilages in vivo. More importantly, this present study was the first to confirm the final fate of implanted allogeneic PB‐MSCs that could survive in cartilage defects for a long time to promote the cartilage repair. Understanding the biological effects and potential molecular mechanisms of KGN in cartilage regeneration will provide insights and platforms for clinical applications as load‐bearing scaffolds.

## 4 Experimental Section

The Ethical Committee of Laboratory Animals of Peking University Third Medical School approved all animal experiment protocols following the Guide for the Care and Use of Laboratory Animals (A2019029).

## Conflict of Interest

The authors declare no conflict of interest.

## Supporting information

Supporting Information

## Data Availability

Research data are not shared.
