# Supplementary material for: Kartogenin‐Conjugated Double‐Network Hydrogel Combined with Stem Cell Transplantation and Tracing for Cartilage Repair
Source: Adv Sci (Weinh). 2022 Oct 17;9(35):2105571. doi: 10.1002/advs.202105571 (PMC9762312; doi:10.1002/advs.202105571)
Supplement: Supplementary file 1 — Supporting Information [file ADVS-9-2105571-s001.pdf]

ADVANCED SCIENCE
Open Access

# Supporting Information

for *Adv. Sci.*, DOI 10.1002/advs.202105571

Kartogenin-Conjugated Double-Network Hydrogel Combined with Stem Cell Transplantation and Tracing for Cartilage Repair

*You-Rong Chen, Xin Yan, Fu-Zhen Yuan, Lin Lin, Shao-Jie Wang, Jing Ye, Ji-Ying Zhang, Meng Yang, De-Cheng Wu*, Xing Wang* and Jia-Kuo Yu**

# Supporting Information

**Kartogenin-Conjugated Double-Network Hydrogel Combined with Stem Cells Transplantation and Tracing for Cartilage Repair**

You-Rong Chen†, Xin Yan†, Fu-Zhen Yuan†, Lin Lin, Shao-Jie Wang, Jing Ye, Ji-Ying Zhang, Meng Yang, De-Cheng Wu*, Xing Wang*, and Jia-Kuo Yu*

† These authors contributed equally to this work.

*Corresponding author (E-mail: yujiakuo@126.com, wangxing@iccas.ac.cn, wudc@sustech.edu.cn)

## Materials and Methods

### Materials

Four-armed poly(ethylene glycol) succinimidyl (4-arm-PEG-NHS, $M_w$ = 10 kDa) and four-armed poly(ethylene glycol) amine (4-arm-PEG-$NH_2$, $M_w$ = 10 kDa) were purchased from SINOPEG, China. Short-chain chitosan (CHI, degree of deacetylation > 90%, viscosity 45 mPas for 1% (w/v) solution, Mw≈10 kPa) was purchased from Jinhu Company, China. Sodium citrate ($Na_3C_6H_5O_7·2H_2O$), sodium hydroxide (NaOH), anhydrous ethanol, 1-ethyl-3-(dimethylaminopropyl) carbodiimide hydrochloride (EDC), N-hydroxysuccinimide (NHS), deuterated water ($D_2O$) and deuterated dimethyl sulfoxide (dDMSO) were purchased from Sinopharm Chemical Reagent Co., Ltd. Shanghai, China. Kartogenin（KGN） was purchased from Selleckchem Co., Houston, TX, USA. Papain, chondroitine sulfate, DAPI, Hoechst33258, calf thymus DNA, 1, 9-Dimethylmethylene blue (DMMB) were purchased from Sigma-Aldrich, Inc., USA. Granulocyte colony-stimulating factor (G-CSF) was acquired from Qilu Pharmaceutical Co. Ltd. Jinan, China. AMD3100 was purchased from MedChemExpress LLC., USA. Ficoll lymphocyte separation medium (1.077 g/mL) were purchased from GE Healthcare, Stockholm，Sweden. RIPA Lysis Buffer (R0010),

proteinase K and 2.5% glutaraldehyde solution were purchased from Solarbio Science & Technology Co., Ltd, Beijing, China. Rhodamine labeled phalloidin were purchased from Cytoskeleton Inc., Denver, USA. Mouse anti-COL-2 primary antibody (CP18) were purchased from Merck Millipore Co., Darmstadt, Germany. Phosphate buffer saline (PBS), 10% neutral formaldehyde solution, hematoxylin dye solution, 1% eosin dye solution, Alexa Fluor® 594 goat anti-mouse IgG antibodies (ZF-0513), goat anti-mouse IgG-HRP secondary antibody (PV-6002) were purchased from ZSGB-BIO Co., Ltd, Beijing, China. Toluidine blue dye solution was purchased from Bioss Biotechnology Co., Ltd. Beijing, China. α-MEM medium, fetal bovine serum, Penicillin-Streptomycin solution, RevertAid First Strand cDNA Synthesis Kit (K1622) and SYBR® Select Master Mix (4472908) were purchased from Thermo Scientific, Carlsbad, CA, USA. Cell Counting Kit-8 assay was purchased from Dojindo Laboratories, Kumamoto, Japan. The Live/Dead Viability Kit assay and TRIZOL reagent were purchased from Invitrogen, Carlsbad, CA, USA. Rabbit Col-2 ELISA kit, IL-1β ELISA kit and TNF-α ELISA kit were provided by Cloud-Clone, Corp., Houston, TX, USA.

**Preparation of PEG, CHI and PEG-CHI DN hydrogels with different CHI contents**

4-arm-PEG-$NH_2$ (400 mg) and 4-arm-PEG-NHS (400 mg) were dissolved in 5 mL of PBS solution, respectively, to prepare precursor solutions 1 and 2. PEG hydrogel was obtained by rapidly mixing precursor solution 1 and precursor solution 2 with a circular oscillometer.

The CHI powder (800 mg) was firstly dissolved in 10 mL of PBS solution, and the saturated sodium citrate solution was added to obtain CHI hydrogel.

CHI (100 mg, 200 mg and 400 mg) was dissolved in 8 mL of PBS solution, and 400 mg of 4-arm-PEG-$NH_2$ was further added to prepare precursor solution 1. 4-arm-PEG-NHS (400 mg) was dissolved in 2 mL of PBS solution to prepare precursor solution 2. After mixing precursor solution 1 and precursor solution 2, PEG-CHI composite hydrogels with CHI solid content of 1 wt%, 2 wt% and 4 wt% were obtained. Then the saturated sodium citrate solution was added on top of the composite hydrogels. After overnight, PEG-CHI DN hydrogels with CHI solid content of 1 wt%, 2 wt% and 4 wt% were obtained, respectively.

**Determination of morphology, pore size and porosity**

A JSM-7900F scanning electron microscope (SEM, Hitachi, Japan) was used to observe the microstructure of the hydrogels. Briefly, the freeze-dried hydrogels were sliced and bonded to the sample stage with conductive resin, then coated with a layer of gold to obtain the SEM images at 5 kV acceleration voltage. The pore size of hydrogels was measured with the help of a measuring tool included in the SEM machine.

The liquid displacement method was used to determine the porosities of freeze-dried hydrogels. First, samples with regular form were calculated and weighed the initial volume and weight ($V_0$ and $W_0$). Second, samples saturated with absolute ethyl alcohol were weighted again ($W_1$). The equation as below was used to calculate the porosities of four hydrogels:

$$\text{Porosity (\%)} = [(W_1 - W_0)/(V_0 \times \rho)] \times 100\%$$

$\rho$ represented the density of absolute ethyl alcohol.

**Compressive testing**

The mechanical properties of hydrogels were calculated with a universal material testing machine of Instron 3365 (Instron Co., USA). Samples (d = 15 mm, h = 10 mm) were prepared and compressive testing was carried out at 3 mm/min. The

fracture strain, compressive stress, and elastic modulus were calculated based on the stress-strain curve.

**Synthesis of KGN conjugated CHI (CHI-KGN)**

Carbodiimide chemistry was utilized to mediate the formation of an amide linkage between the terminal carboxylic group of KGN and the amine group of CHI. Briefly, EDC/NHS solution at the appropriate concentration and molar ratio were prepared in ionized water according to the manufacturer's instructions. KGN was immersed in an appropriate concentration mixture of EDC and NHS for 1 h at 25 °C. CHI was reacted with the NHS-esterified KGN for 24 h with low-speed stirring. The KGN conjugated CHI (CHI-KGN) was then dialyzed against deionized water for 72 h and further lyophilized to obtain a purified material.

**Characterization of CHI, KGN and CHI-KGN conjugate**

$^1$H NMR (proton nuclear magnetic resonance) spectroscopy was used to characterize the structures of CHI, KGN and CHI-KGN conjugate. The chemical shifts were measured in parts per million (ppm, d) using $D_2O$ or DMSO-$d_6$ as the internal reference. $^1$H NMR spectra were obtained by an AVANCE 400 NMR spectrometer (Bruker BioSpin, Rheinstetten, Germany) at room temperature.

**Preparation of PEG-CHI-KGN composite hydrogel and DN hydrogel**

Functionalized PEG-CHI-KGN composite hydrogel and DN hydrogel with CHI-KGN content of 2 wt% were prepared by in-situ and/or ionic crosslinking gel formation. CHI-KGN conjugate (200 mg) was dissolved in 8 mL of PBS solution, and 400 mg of 4-arm-PEG-$NH_2$ was further added to prepare precursor solution 1. 4-arm-PEG-NHS (400 mg) was dissolved in 2 mL of PBS solution to prepare precursor solution 2. After mixing precursor solution 1 and precursor solution 2, PEG-CHI-KGN composite hydrogels with CHI-KGN content of 2 wt% were obtained. The gelation

time of composite hydrogels was recorded by the vial tilting method. Then the saturated sodium citrate solution was added to the composite hydrogels. After overnight, PEG-CHI-KGN DN gel with CHI-KGN content of 2 wt% was obtained.

**Rheological measurements**

Thermo Haake Rheometer with a cone-parallel plate geometry (d = 35 mm) was used to measure the shear modulus of PEG-CHI and PEG-CHI-KGN composite hydrogels and DN hydrogels. Frequency sweep experiments were performed with a constant strain of 0.05% in the frequency range of 0.1-100 rad $s^{-1}$. The hydrogels (d = 35 mm, h = 3.5 mm) were placed between two plates and tested at a gap of 3 mm.

**Cyclic compressive loading test**

To reveal the energy dissipation capacity of the PEG-CHI, PEG-CHI-KGN composite hydrogels and DN hydrogels, we compared the stress-strain curves of these four hydrogels by using the cyclic loading experiments at the rate of 3 mm $min^{-1}$. Samples (d = 15 mm, h = 7.5 mm) were prepared and a cyclic compressive loading test was carried out with a universal material testing machine of Instron 3365 (Instron Co., USA). The hydrogel samples were compressed to the 60% strain of the gel and then restored to the original height at the same rate, thereby obtaining the load-unload curve. The area between the curves was the energy dissipated during cyclic compression test.

**Swelling properties**

The swelling properties of hydrogels were measured by preparing the dried hydrogel samples, recording the initial weight ($W_d$), and then placing the samples in 50 mL of PBS (pH 7.4) at 37 °C. The samples were weighed again ($W_s$) after incubation for a certain time (0 h, 0.5 h, 1 h, 2 h, 4 h, 6 h, 8 h, 12 h, 16 h, 24 h, 48 h, 72 h, 96 h and 120 h). The calculational equation of swelling ratio was used as below:

$$\text{Swelling ratio (\%)} = (W_s - W_d)/W_d \times 100\%$$

**Degradation *in vitro***

The weight loss of samples in PBS solution with and without proteinase K (6 U/mL) were recorded to describe the degradation behaviors of hydrogels. We recorded the initial weight of lyophilized samples ($W_0$), and incubated them in PBS solution at 37 °C. After incubating for a certain time (1 d, 4 d, 7 d, 14 d, 21 d, 28 d, 35 d and 42 d), the samples were lyophilized and weighed again ($W_t$). The degradation ratio can be calculated according to the following equation:

$$\text{Degradation ratio (\%)} = (W_0 - W_t)/W_0 \times 100\%$$

**Cumulative release of KGN *in vitro***

The samples were placed in PBS solution (pH 7.4, 2.5 mL) with and without proteinase K (6 U/mL) at 37 °C under continuous agitation to obtain the release behavior of KGN from the PEG-CHI-KGN DN hydrogels. After incubation for a certain time (1 d, 3 d, 5 d, 7 d, 10 d, 14 d, 21 d, 28 d, 35 d, and 42 d), we withdrew the PBS solution with released KGN followed by adding the same volume of fresh PBS to maintain the total volume of 2.5 mL. The released amounts of KGN were quantified by a standard curve method on UV−vis spectrophotometry, and the absorption peak of KGN was at 279 nm.[1] Cumulative release rate of KGN (%) = (KGN cumulative release/total KGN in hydrogels) ×100%

**Isolation and culture of PB-MSCs**

Ethical Committee of Laboratory Animals of Peking University Third Medical School approved all animal experiment protocols following the Guide for the Care and Use of Laboratory Animals. Peripheral blood (PB, 20 mL) was isolated from the central auricular arteries of New Zealand White rabbits (2 months, male, 1 kg) after mobilizing with granulocyte colony-stimulating factor (G-CSF, Qilu Pharmaceutical

Co. Ltd.) and AMD3100 (MedChemExpress LLC., USA). Peripheral blood mononuclear cells (MNCs) were collected using the method of density gradient centrifugation and cultured in a medium of a-MEM with 15% fetal bovine serum, 100 U/mL penicillin and 100 U/mL streptomycin. The culture medium was replaced every 3 days until the confluence of primary PB-MSCs reached around 90%, and then subculture was carried out at a ratio of 1:3. PB-MSCs at passage 3 [PB-MSCs (P3)] were used for subsequent experiments.[2]

**Tri-lineage differentiation potential of PB-MSCs**

Multilineage differentiation potential of PB-MSCs was assessed with the method of tri-lineage differentiations. Osteogenic medium, adipogenic induction and maintenance medium, and chondrogenic medium were used for osteogenesis, adipogenesis and chondrogenesis of PB-MSCs, respectively. The deposition of calcium nodules, accumulation of lipid vacuoles in cells and cartilage-specific aggregating proteoglycans were detected with Alizarin red staining, Oil red O staining, and Alcian blue staining.

**Cytotoxicity studies of hydrogels**

Cell Counting Kit-8 assay (CCK-8, Dojindo Laboratories, Japan) was applied to evaluate the cytotoxicity of the hydrogel by culturing PB-MSCs with the extracting liquid of hydrogels. Rabbit PB-MSCs at passage 3 were seeded into 96-well microplates ($8 \times 10^3$ cells/100 μL/well), and then 10 μL of hydrogel extracting liquid or PBS was added into the cell medium and further incubated for a predetermined time. After incubation for 24 h and 48 h, we removed the original culture medium and added a fresh culture medium (100 μL) with CCK-8 reagent (10 μL). A microplate reader (Thermo, USA) was used to obtain the optical density (OD) value of 450 nm

after incubating for another 1 h. The equation below was used to calculate the cell viability:

$$\text{Cell viability} = [(As-Ab)/(Ac-Ab)] \times 100\%$$

Where the As, Ac, Ab are the optical density (OD) of hydrogels for the extracts group, control group and blank group. It was considered cytotoxic if cell viability was < 70% after incubation with hydrogel extracting liquid.[3]

**Cell seeding and cell-hydrogel construct culture**

50 μL of PB-MSCs suspension ($1 \times 10^7$ cells/mL) was carefully seeded on freeze-dried hydrogel (d = 5 mm, h = 1.5 mm) with the method of centrifugation as we previously reported.[4] The cell-hydrogel composites were incubated for 1 h to facilitate cell adhesion, and then 2.5 mL of fresh chondrogenic differentiation medium (removal of TGF-β3 components, Cyagen Biosciences Inc., Suzhou, China) was added for further culture. The culture medium was replaced every 3 days until cell-hydrogel composites were used in subsequent experiments.

**Cell proliferation on hydrogels**

Cell proliferation on hydrogels was assessed by using the method of CCK-8 assay. After incubation for 1, 4 and 7 days, we removed the original culture medium and added a fresh culture medium (500 μL) with CCK-8 reagent (50 μL). After incubating for another 0.5 h-1 h, 110 μL of liquid from each sample was transferred to a new 96 well plate, and a microplate reader (Thermo, USA) was used to obtain the optical density (OD) value of 450 nm.

**Cell viability**

LIVE/DEAD Viability/Cytotoxicity Kit assay (Invitrogen, CA, USA) was used to visualize the survival of PB-MSCs on hydrogels. After culturing for 7 days, the cell-hydrogel composites were washed with PBS solution to remove the culture

medium and immersed in reagents of calcein AM (2 mM) and ethidium homodimer-1 (4 mM) for 1 h at 37 ℃. Live (green) and dead (red) cells were detected using confocal microscopy with an excitation wavelength of 568 nm and 488 nm.

**Analysis of fibrotic, cartilage-specific and hypertrophic genes expression**

After incubation for 7 and 14 days, cell-hydrogel composites were removed from the culture medium and washed with PBS. TRIZOL reagent (Invitrogen, Carlsbad, CA, USA) and RevertAid First Strand cDNA Synthesis Kit (K1622, Thermo Scientific, Carlsbad, CA, USA), following the manufacturer's instructions, were used to extract total RNA and reverse-transcribe isolated RNA, respectively. According to the conditions reported in previous literature, quantitative Real-time polymerase chain reaction (RT-PCR) analysis was performed to detect the expression of the fibrotic marker gene (*COL1A1*), cartilage-specific marker gene (*COL2A1* and aggrecan, *ACAN*) and hypertrophic marker gene (collagen type-10, *COL-10*) by using an ABI 7300 real-time PCR system (Applied Biosystems, Foster City, CA, USA) with SYBR Select Master Mix (4472908, Thermo Scientific, Carlsbad, CA, USA). The value of a relative expression in these target genes was plotted as $2^{-\triangle\triangle CT}$ with the previous method.[5] The PCR primers are listed in Table S1.

**Table S1.** Primer sequences used for real-time PCR

| Gene | Forward primers (5'−3') | Reverse primers (5'−3') |
|---|---|---|
| *COL1A1* | TGGCAAGAACGGAGATGACG | GCACCATCCAAACCACTGAA |
| *COL2A1* | CCACGCTCAAGTCCCTCAAC | AGTCACCGCTCTTCCACTCG |
| *ACAN* | CGTGGTCTGGACAGGTGCTA | GGTTGGGGTAGAGGTAGACG |
| *COL-10* | AAGTGGACCGAAAGGAGACA | TGGAAACCCATTCTCACCTC |
| *GAPDH* | CCATCACCATCTTCCAGGAG | GATGATGACCCTTTTGGCTC |

*Abbreviations: COL1A1*: collagen type-1; *COL2A1*: collagen type-2; *ACAN*: aggrecan; *COL-10*: collagen type-10; *GAPDH*: glyceraldehyde-3-phosphate dehydrogenase

**Biochemical assessment**

Hoechst33258 staining and the fluorometric assay were performed to measure the DNA content of cell-hydrogel composites.[6] After culturing for 1, 7 and 14 days, the cell-hydrogel composites were weighed and then digested in a prepared papain solution (Sigma, St. Louis, Missouri, USA) at 60 °C for 24 h to obtain aliquots of the sample digestion. Aliquots of the sample digestion (10 μL) were mixed with Hoechst33258 working solution (2 μg/mL, 100 μL) and incubated at 37 °C for 1 h. A microplate reader (Thermo, USA) was used to detect the fluorescent intensities with excitation wavelength of 360 nm and emission wavelength of 460 nm. A standard curve of calf thymus DNA (Sigma, St. Louis, Missouri, USA) was used to normalize the content of DNA.

High efficient RIPA tissue/cell lysis solution (R0010, Solarbio Science & Technology Co., Ltd., Beijing, China) was used to obtain the total proteins of the cell-hydrogel constructs. Rabbit COL-2 ELISA Kits (Cloud-Clone, Corp., Houston, TX, USA) was applied to measure the COL-2 content according to the manufacturing protocol.

For glycosaminoglycans (GAG) assay, 10 μL of the digested sample was stained at 37 °C for 30 min with 100 μL of 1,9-dimethylmethylene blue (DMMB; Sigma, St. Louis, MO, USA). The optical density (OD) was measured at 525 nm. A standard curve based on chondroitin-6-sulfate from shark (Sigma, St. Louis, MO, USA) was used to determine the GAG content.[7]

**Immunofluorescent staining of COL-2**

Immunofluorescent staining was performed to visualize the secretion of COL-2 in cell-hydrogel composites after culturing for 14 days. First, cell-hydrogel composites were washed with PBS to remove the culture medium and fixed with 4% paraformaldehyde. Then, 10% bovine serum albumin (BSA), mouse anti-COL-2 primary antibody (CP18, Merck KGaA, Darmstadt, Germany), Alexa Fluor® 594 goat anti-mouse IgG antibodies (ZF-0513, ZSGB-BIO Co., Ltd., Beijing, China) and DAPI (Sigma, St. Louis, Missouri, USA) were used successively to incubate composites, and the immunofluorescence images were obtained by using confocal microscopy. The fluorescent intensity of COL-2 was calculated as previously reported.[8]

**Cell morphology on scaffolds**

We performed cytoskeleton staining and used confocal microscopy to observe the morphology of PB-MSCs in hydrogels on day 14. After removal of culture medium of scaffolds and fixing with paraformaldehyde, rhodamine phalloidin (100 nM; Cytoskeleton Inc., Denver, USA) and DAPI working solution (1 μg/mL, Sigma-Aldrich, Inc., USA) stained the cytoskeleton (30 min) and nuclei (5 min) at 37 ℃, respectively.

**Animal model assessments**

Adult female New Zealand white rabbits weighing 3.0-3.5 kg (about 6-9 months) were used for the study *in vivo*. 95 rabbits were randomized into five groups (two knees of each rabbit were used): Blank group, MSCs only group, PEG-CHI group, PEG-CHI-KGN group and Sham group. The trochlear groove of femur was exposed after anesthesia (pentobarbital sodium, i.v., 30 mg/kg), fixation, skin disinfection, and incision of skin, subcutaneous tissue and articular capsule. The cartilage defect (5 mm in diameter and 2 mm in depth) was created by the modified corneal trephine in the

trochlear groove of the distal femur. In the Blank group, the cartilage defects were left empty and served as the control. In the MSCs only group, the cartilage defects were implanted with allogenic PB-MSCs ($1\times10^6$ cells, from the male rabbits). In the PEG-CHI and PEG-CHI-KGN groups, the cartilage defects were implanted with PEG-CHI DN hydrogels or PEG-CHI-KGN DN hydrogels containing allogenic PB-MSCs ($1\times10^6$ cells, from the male rabbits). The lyophilized hydrogel scaffolds were sterilized by UV light, and cell-hydrogel complexes were prepared in advance with the previously reported centrifugation method.[4] In the Sham group, after exposing the trochlea of the femur, the wound was immediately sutured without making cartilage defects as a control. After the operation, all rabbits were treated with antibiotic prophylaxis (penicillin sodium, i.m., 100000 U/kg, qd, 3d) and analgesia (meloxicam injection, 0.3 mg/kg, s.c., qd, 3d), and allowed to move freely in their single cages. 4, 8 and 12 weeks later, rabbits were sacrificed for further study.

**Cartilage magnetic resonance imaging**

At 4, 8 and 12 weeks post-surgery, each rabbit in experimental groups underwent MRI analysis for evidence of new cartilage growth and knee effusion on a Siemens TIM Trio 3T MRI scanner (Siemens, Erlangen, Germany) using a small animal-specific knee coil (Chenguang Medical Technologies Co., Ltd, Shanghai, China) to improve the signal-to-noise and contrast-to-noise ratios. The protocol included three sequences for morphological observation and quantitative analysis; total acquisition time was ca. 20 min. MRI examination imaging sequences are listed in Table S2.

**Table S2.** MRI examination imaging sequences

| Series description | Plane | TR (ms) | TE (ms) | FOV (mm) | Flip angle | Slice thickness (mm) | Distance factor (%) | Matrix | BW (Hz/pixel) |
|---|---|---|---|---|---|---|---|---|---|

| | | | | | | | | | |
|---|---|---|---|---|---|---|---|---|---|
| Fat-saturated T2-weithted TSE imaging | Sagittal | 2000 | 72 | 70 | 150 | 2 | 10 | 256×256 | 199 |
| PD-weighted TSE imaging | Sagittal | 2000 | 36 | 70 | 150 | 2 | 10 | 256×256 | 199 |
| 3D T1-weighted spoiled GRE imaging | Sagittal | 26 | 4.6 | 60 | 25 | 1 | 20 | 512×256 | 200 |

*Abbreviations:* TR, time of repetition; TE, time of echo; FOV, the field of view; BW, bandwidth; 3D, 3 dimensions; TSE, turbo spin echo; PD, proton density; GRE, gradient echo.

**Synovial fluid collection and analysis**

At 1, 2, 4, 8 and 12 weeks post-surgery, synovial fluid was collected via a 2-mL syringe with an 18-gauge needle. The supernatants were assayed for interleukin 1β (IL-1β) and tumor necrosis factor α (TNF-α) utilizing standard enzyme-linked immunosorbent assay (ELISA) kits.

**Observation of repaired cartilage defects**

According to the criteria of the International Cartilage Repair Society (ICRS) macroscopic evaluation of cartilage repair (Table S3), a gross examination was performed for each knee to evaluate defect filling, tissue integration and surface smoothness.[9]

**Table S3.** ICRS macroscopic evaluation of cartilage repair

| Cartilage repair assessment ICRS | Score |
|---|---|
| A) Degree of defect repair | |
| In level with surrounding cartilage | 4 |
| 75% repair of defect depth | 3 |
| 50% repair of defect depth | 2 |
| 25% repair of defect depth | 1 |

| | |
|---|---|
| 0% repair of defect depth | 0 |
| B) Integration to border zone | |
| Complete integration with surrounding cartilage | 4 |
| Demarcating border <1 mm | 3 |
| 3/4th of graft integrated, 1/4th with a notable border > 1 mm width | 2 |
| 1/2 of graft integrated with surrounding cartilage, 1/2 with a notable border >1 mm | 1 |
| From no contact to 1/4th of graft integrated with surrounding cartilage | 0 |
| C) Macroscopic appearance | |
| Intact smooth surface | 4 |
| Fibrillated surface | 3 |
| Small, scattered fissures or cracks | 2 |
| Several, small or few but large fissures | 1 |
| Total degeneration of a grafted area | 0 |
| Overall repair assessment | |
| Grade I: normal | 12 |
| Grade II: nearly normal | 11-8 |
| Grade III: abnormal | 7-4 |
| Grade IV: severely abnormal | 3-1 |

**Histological analysis**

At 4, 8 and 12 weeks post-surgery, the repaired knees were harvested, fixed in 10% neutral buffered formaldehyde (pH 7.4) for 72 h at room temperature, decalcified in 8.8% formic acid solution with 4% neutral buffered formaldehyde at 4 ℃. The decalcified samples were trimmed, dehydrated in a graded ethanol series, and embedded in paraffin. Serial sections (5-μm thick) through the center of the repair site were cut and stained with hematoxylin & eosin (HE) and toluidine blue (TB). According to standard protocols, immunohistochemistry (IHC) was performed using primary antibodies against type II collagen (CP18) according to standard protocols.

The Wakitani histological scoring system was used to quantitatively evaluate the repair of articular cartilage defects (Table S4).[10]

**Table S4.** Histological scoring system for evaluation of repair of cartilage defects

| 1) Features of repair cartilage | Score |
|---|---|
| A) Cell morphology | |
| Hyaline cartilage | 6 |
| Mostly hyaline cartilage> 3/4 | 5 |
| Partly hyaline cartilage1/4–3/4 | 4 |
| Mostly fibro-cartilage> 3/4 | 3 |
| Partly fibro-cartilage1/4–3/4 | 2 |
| Mostly non-cartilage | 1 |
| None cartilage only | 0 |
| B) Matrix staining (metachromasia) | |
| Normal (compared to host) | 4 |
| Slightly reduced staining > 3/4 | 3 |
| Moderately reduced staining 1/4–3/4 | 2 |
| Remarkably reduced staining < 1/4 | 1 |
| No metachromatic staining | 0 |
| C) Surface regularity | |
| Smooth > 3/4 | 3 |
| Moderate 1/2–3/4 | 2 |
| Irregular 1/4–1/2 | 1 |
| Severe irregular | 0 |
| D) Thickness of the defect | |
| Normal > 2/3 | 2 |

| | |
|---|---|
| Moderate 1/3–2/3 | 1 |
| Thin < 1/3 | 0 |
| E) Integration of repair tissue to the surrounding articular cartilage | |
| Both edges integrated | 2 |
| One edge integrated | 1 |
| Both edges not integrated | 0 |
| F) Arrangement of repair cartilage | |
| Column-like arrangement | 2 |
| Partly column-like arrangement | 1 |
| Disordered | 0 |
| 2) Features of surrounding tissue | |
| G) Remodeling of subchondral bone | |
| Complete reconstruction | 3 |
| Continuous but incomplete reconstruction | 2 |
| Discontinuous, greater than 50% reconstruction | 1 |
| Discontinuous, less than 50% reconstruction | 0 |
| H) Effect on adjacent cartilage | |
| Normal (compared to host) | 3 |
| Slightly reduced staining > 3/4 | 2 |
| Remarkably reduced staining 1/4–3/4 | 1 |
| Little or no metachromatic staining < 1/4 | 0 |

**SEM observation of cartilage repair**

At 12 weeks after the operation, SEM was used to assess microscopic changes in the surfaces of the repaired areas. Briefly, samples were trimmed without disturbing the cartilage surface, fixed immediately in 5 mL of 2.5% glutaraldehyde for 24 h at 4 ℃,

dehydrated in a graded ethanol series, and subjected to critical point drying to ensure complete dehydration. The surface of the knees was coated with a 5 nm layer of gold in a high-vacuum gold spatter coater, and then viewed using high-resolution SEM images (JSM-7900F; Hitachi Ltd., Tokyo, Japan).

**Nanoindentation assessment of repaired tissue**

Biomechanical analysis of repair tissues was performed using nanoindentation. Samples were isolated from the central part of repaired tissues. Hydration was maintained utilizing a circumfluent PBS solution at room temperature. All indentations were performed using a displacement-controlled PIUMA Nanoindenter (Optics11 B.V., Amsterdam, The Netherlands) with a 38.5-μm or 46.5-μm radius spherical probe tip. A trapezoidal load function was applied to each indent site with loading (2 s), hold (1 s), and unloading (2 s). Indentations were force-controlled to a maximum indentation depth of 10 μm. The effective Young's modulus of the repaired tissue was calculated according to the slope analysis of the stress-strain curve.[11]

**Fluorescent in situ hybridization (FISH) detection**

To detect PB-MSCs in the regenerated tissue of PEG-CHI-KGN group, we performed a FISH assay on three samples from each time point according to the protocol provided by Shanghai Gefan Biotechnology Co., Ltd. (Shanghai, China)[12] using FAM labeled 45-bp oligonucleotide mRNA probe (5’-FAM-TCACTTTACGTGTTCCTCCTGTATTGCACTGGTGGTTTGTGCTAA-3’) built on the oryctolagus cuniculus sex determining region Y (SRY) gene sequence (GenBank: AY785433.1). Tissues were examined with a fluorescent confocal microscope (Leica Microsystems, Heidelberg, Germany) and images were recorded. To assess the specificity and effectiveness of the probe, male and female rabbits' knee joint cartilage were served as positive and negative controls, respectively.

**RNA sequencing**

The regenerated cartilage of the Blank group, MSCs only group, PEG-CHI group, PEG-CHI-KGN group and Sham group were collected 12 weeks after surgery, after which total RNA was isolated as described previously.[13] RNA sequencing (RNA−seq) was then carried out via a commercially available service (BGI, Huada Gene, Wuhan, China). Briefly, after total RNA was fragmented into short fragments and mRNA was enriched using oligo (dT) magnetic beads, followed by cDNA synthesis. Double-stranded cDNA was purified and enriched by PCR amplification, after which the library products were sequenced using BGIseq-500. The KEGG pathway and GO bioinformatics analyses were performed by the BGI, using the Dr. TOM approach, an in-house customized data mining system of the BGI. Altered (upregulated or downregulated) expression of genes was expressed as log2FC, which represents log-transformed fold change (log2FC = log2[B]−log2[A], while A and B represent values of gene expression for different treatment conditions). The significant levels of terms and pathways were corrected by Q value with a rigorous threshold (Q value ≤ 0.05) by Bonferroni. Utilizing Cytoscape MCODE plug-ins, process activity networks were created for enriched biological pathways with significance ($P<0.05$), including upregulated and downregulated genes. By measuring the Pearson correlation coefficient between genes, it was possible to determine the degree and K-core values of each substantially DEG. The interactions of gene clusters with the highest scores are also shown in the article.

**Quantitative RT-PCR validation for selected genes in RNA-Seq results**

To confirm the accuracy of RNA-Seq results, we selected the most critical gene cluster (*AURKB, DLGAP5, CDCA8, BUB1B, BUB1 and CCNB2*) for qRT-PCR validation from the enriched signaling pathways according to KEGG analysis. qRT-PCR was performed in the Analysis of Fibrotic, Cartilage-Specific and Hypertrophic Genes Expression section. The primers for the quantification of these genes were listed in Table S5. The relative expression level of mRNA for each gene was calculated using GAPDH as an endogenous reference gene using the $2^{-\Delta\Delta Ct}$ method.

**Table S5.** Primer sequences of genes selected from RNA-seq for validation

| Gene | Forward primers (5'−3') | Reverse primers (5'−3') |
|---|---|---|
| *AURKB* | CGGCAGGTTCGGTAGTTCC | GGAGCGGTTCAAGAGGACAA |
| *DLGAP5* | TCAAAGGCAAACGACCAAGC | GTTCTGGCAACCTGCTTTGC |
| *CDCA8* | ACAAATGAGAAGGCGGCAGA | AGTCCCTTTGTCCACGTTCC |
| *BUB1B* | TTTAGCCCAACGAAGACCCC | CTCACTCAGGGGCTCGATTC |
| *BUB1* | TCAGTCCAATCTCACGGCAC | GAGAGCTTGGGGCATCACTT |
| *CCNB2* | TGGACGTATGCGTGCTATCC | CTTCTTGCGGGAGACTGGTT |
| *GAPDH* | CCATCACCATCTTCCAGGAG | GATGATGACCCTTTTGGCTC |

*Abbreviations: AURKB*: Aurora kinase B; *DLGAP5*: DLG associated protein 5; *CDCA8*: cell division cycle associated 8; *BUB1B*: BUB1 mitotic checkpoint serine/threonine kinase B; *BUB1*: BUB1 mitotic checkpoint serine/threonine kinase; *CCNB2*: cyclin B2; *GAPDH*: glyceraldehyde-3-phosphate dehydrogenase

**Immunofluorescent staining of selected genes expression in RNA-Seq results**

Immunofluorescent staining was performed to visualize the secretion of AURKB, DLGAP5, BUB1B, BUB1, CCNB2 and CDCA8 in PB-MSCs after culturing for 14

days with or without KGN. First, PB-MSCs were washed with PBS to remove the culture medium and fixed with 4% paraformaldehyde. Then, 10% bovine serum albumin (BSA), primary antibody (ab3609/TA500533S/sc-365685/67726-1-Ig/ab279368/sc- 377004), Alexa Fluor® 594 goat anti-mouse IgG antibodies (ZF-0513, ZSGB-BIO Co., Ltd., Beijing, China) and DAPI (Sigma, St. Louis, Missouri, USA) were used successively to incubate PB-MSCs, and the immunofluorescence images were obtained by using confocal microscopy. The fluorescent intensity of proteins was calculated as previously reported.[8]

**Immunohistochemical staining to detect the genes expression of regenerated cartilage**

At 12 weeks post-surgery, the repaired knees were harvested, fixed in 10% neutral buffered formaldehyde (pH 7.4) for 72 h at room temperature, and decalcified in 8.8% formic acid solution with 4% neutral buffered formaldehyde at 4 ℃. The decalcified samples were trimmed, dehydrated in a graded ethanol series, and embedded in paraffin. Serial sections (5-μm thick) through the center of the repair site were cut and stained with immunohistochemistry. Immunohistochemistry was performed using primary antibody (ab3609/TA500533S/sc-365685/67726-1-Ig/ab279368/sc-377004) according to standard protocols. Image J was used to semi-quantitatively determine the protein expression intensity.

**Statistical analysis**

We adopted Shapiro–Wilk approach for normality test and Levene’s test method for homogeneity of variance. The data met normal distribution were shown as means ± standard deviation (SD). Student’s t test was used to compare the normally distributed

data between two groups, and one-way ANOVA with Tukey's post-hoc test was used to calculate the differences of such data among three or more groups. Non-normal distribution data were described by medians (interquartile range), and analyzed by the Wilcoxon rank sum test. The qualitative data were reported as number and analyzed by chi-square test. Sample size (n) for each statistical analysis was displayed in the corresponding figure. A *P value* < 0.05 (two-sided) was considered statistically significant. All analyses were carried out using SPSS 25.0 software (SPSS Inc., Armonk, USA).

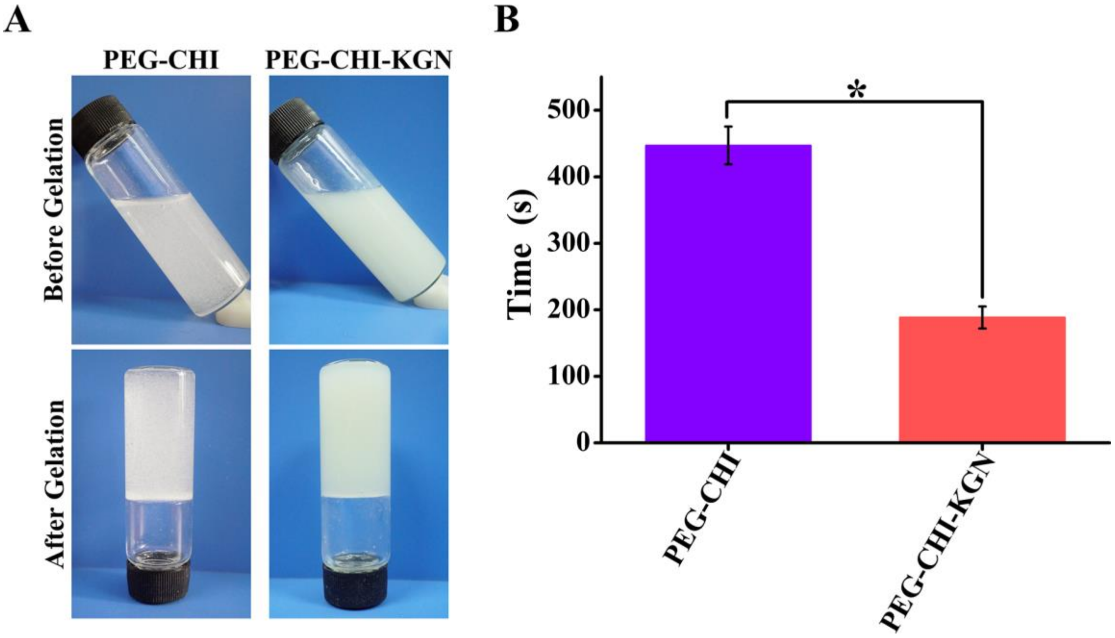


**Figure S1.** (A) Gelation process of PEG-CHI and PEG-CHI-KGN composite hydrogels. (B). Gelation time of PEG-CHI and PEG-CHI-KGN composite hydrogels (n = 5, *$P$ < 0.05).

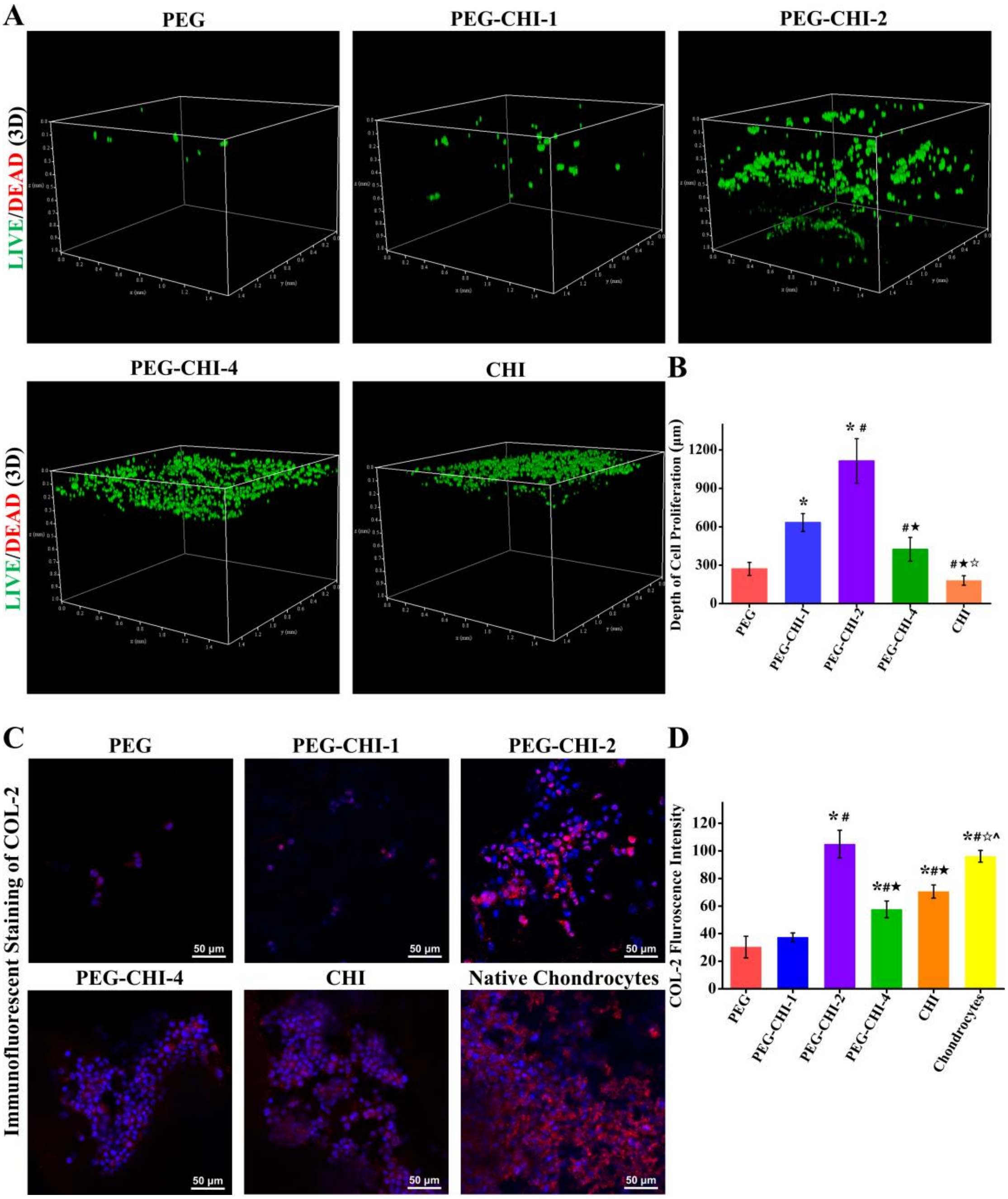


**Figure S2.** (A) 3D renderings of PB-MSCs to show cell distribution in hydrogels after 3 days of culture *in vitro*. (Green: live cells, Red: dead cells). (B) Distribution depth of PB-MSCs in five hydrogels (n = 5). (C) Immunofluorescent staining to visualize the production of COL-2 in the PB-MSCs-hydrogel composites and native chondrocytes after 14 days of *in vitro* culture (Blue: DAPI, Red: COL-2). (D) Fluorescence intensity of COL-2 in the PB-MSCs-hydrogel composites (n = 3). (*$P$ <

0.05 vs. PEG group, $^{\#}P < 0.05$ vs. PEG-CHI-1 group, $^{\star}P < 0.05$ vs. PEG-CHI-2 group, $^{\star}P < 0.05$ vs. PEG-CHI-4 group, ^P < 0.05 vs. CHI group)

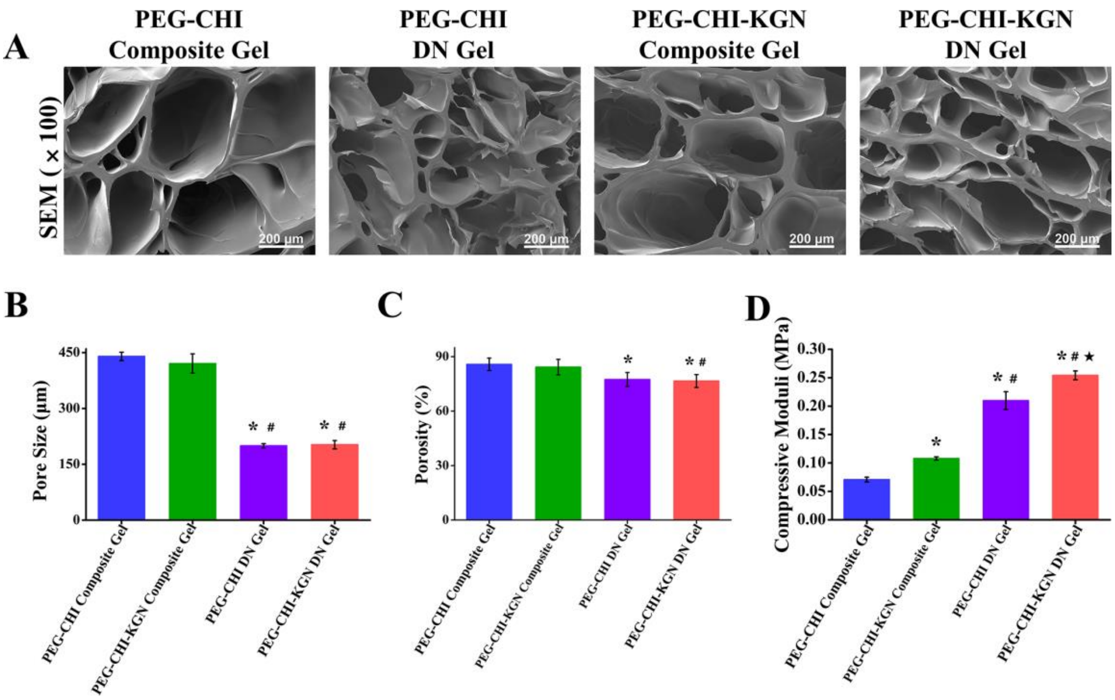


**Figure S3.** (A) SEM images of PEG-CHI and PEG-CHI-KGN composite and DN gels. (B) The average pore sizes of four hydrogels (n = 3). (C) The porosities of four hydrogels (n = 5). (D) Compressive moduli of four hydrogels (n = 3). ($^{*}P < 0.05$ vs. PEG-CHI Composite Gel, $^{\#}P < 0.05$ vs. PEG-CHI-KGN Composite Gel, $^{\star}P < 0.05$ vs. PEG-CHI DN Gel)

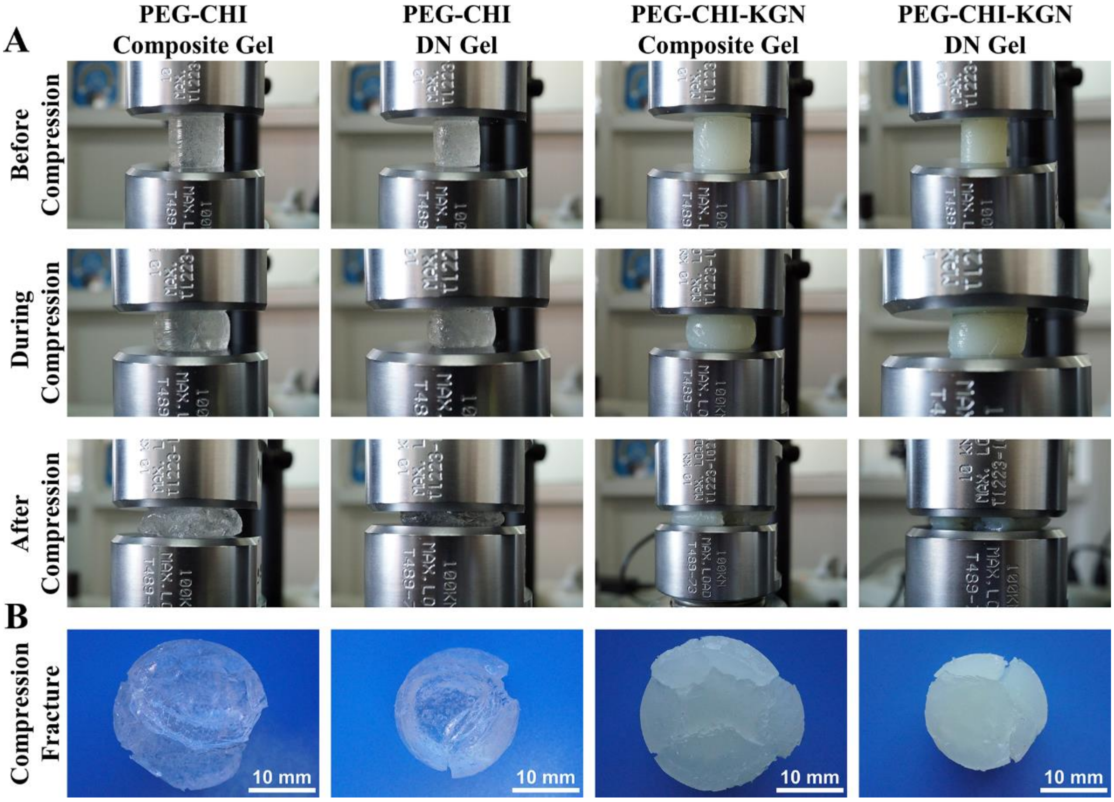


**Figure S4.** (A) The process of compression test and (B) Morphologies of hydrogels during fragmentation.

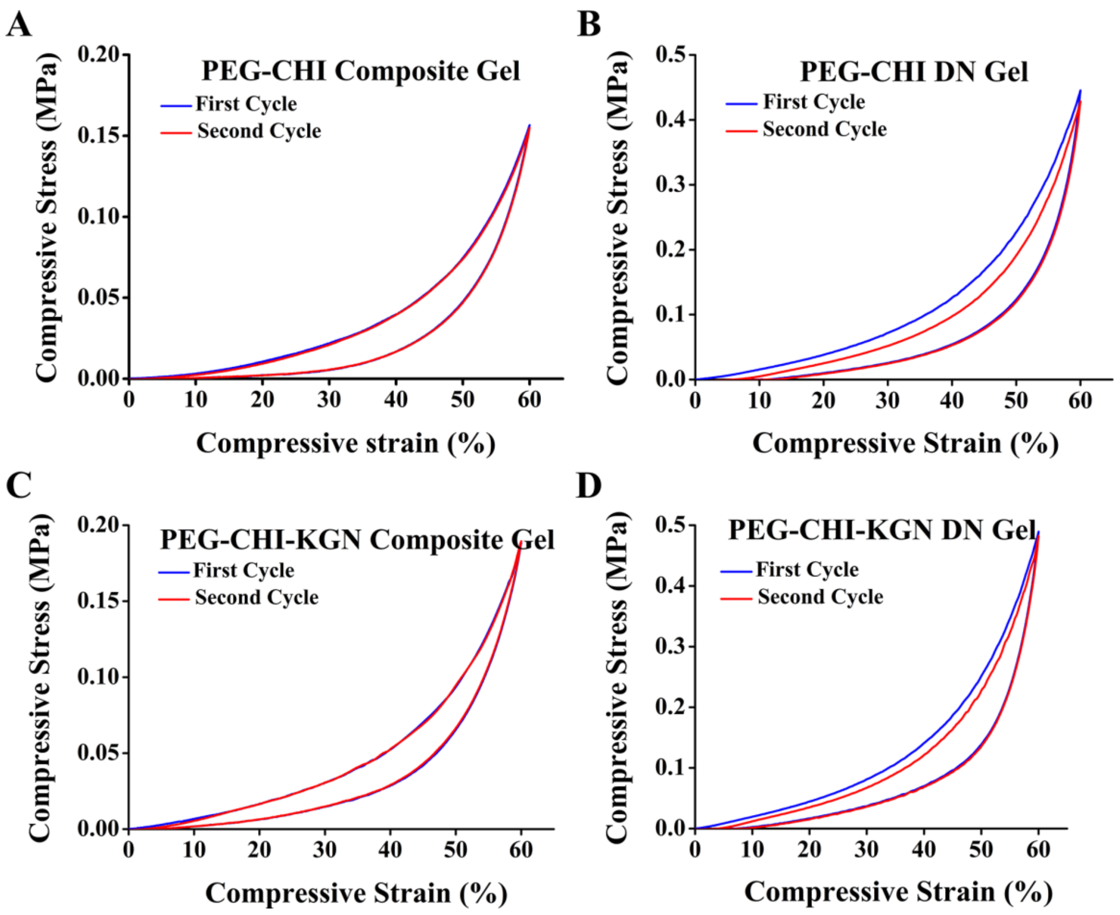


**Figure S5.** Cyclic loading experiments of composite and DN gels to 60% strain. (A) PEG-CHI composite gel, (B) PEG-CHI DN gel, (C) PEG-CHI-KGN composite gel, and (D) PEG-CHI-KGN DN gel.

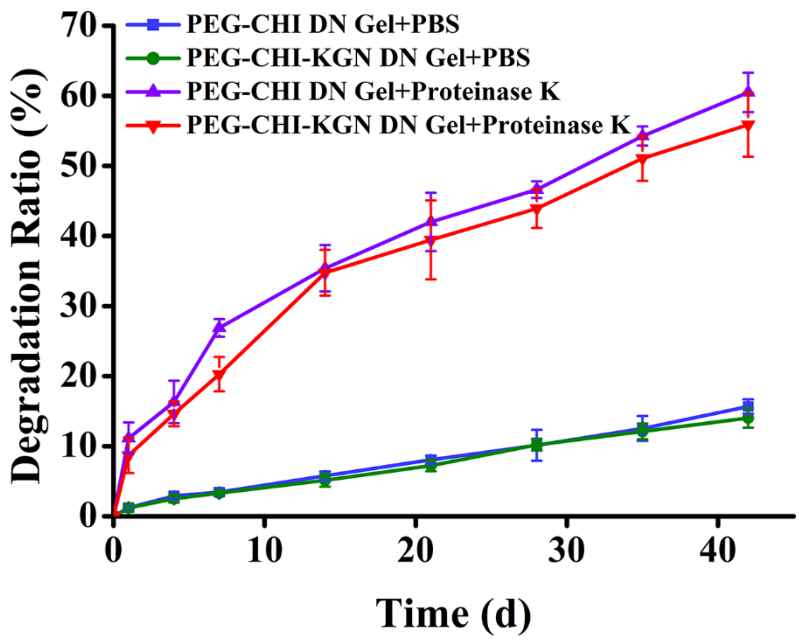

**Figure S6.** *In vitro* degradation of hydrogels in PBS solution with or without protease K at each time point (n = 5).

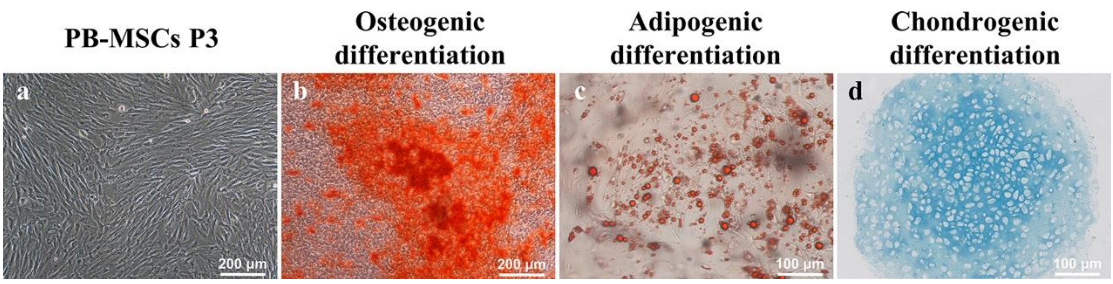


**Figure S7.** The morphological characteristics and multilineage differentiation potential of PB-MSCs. (a) PB-MSCs showed a typical spindle shape at passage 3 *in vitro* (×100). (b) Osteogenic differentiation was assessed by Alizarin red staining. (c) Adipogenic differentiation was detected by Oil Red O staining. (d) Chondrogenic differentiation was confirmed by Alcian Blue staining.

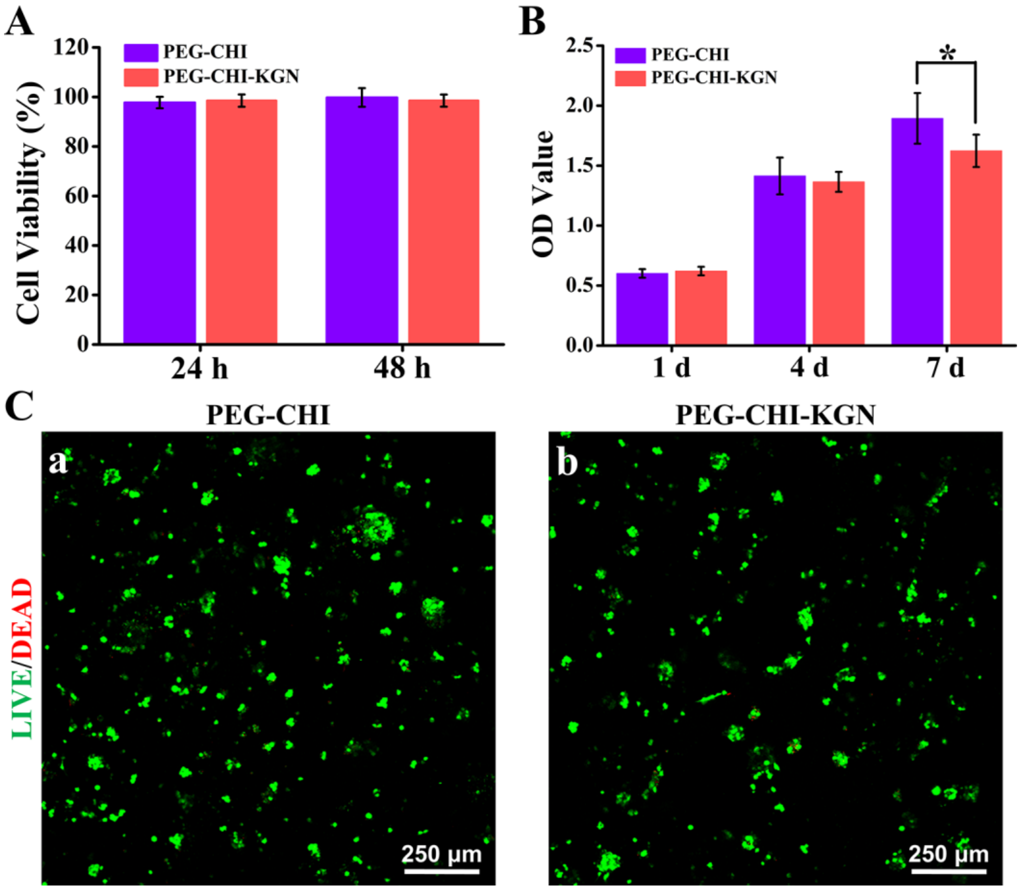

**Figure S8.** (A) Detection of cytotoxicity of hydrogels using CCK-8 assay (n = 5, *P* > 0.05). (B) CCK-8 assay detected the cell proliferation of four groups over time (n = 5, **P* < 0.05). (C) Cell viability of four groups in hydrogels was demonstrated by using Live/Dead staining after 7 days of culture *in vitro*. Green represented live cells and red represented dead cells.

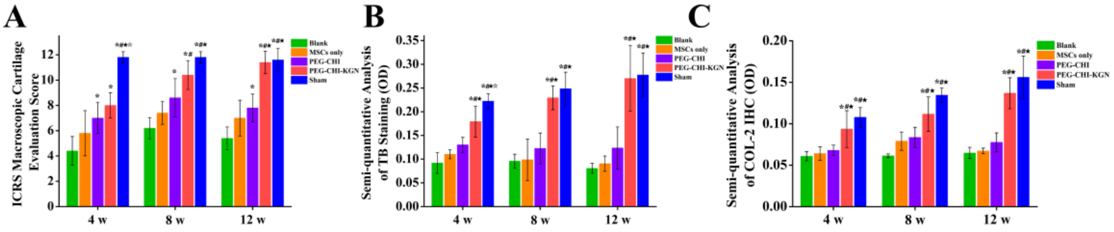


**Figure S9.** (A) International cartilage repair society (ICRS) macroscopic evaluation of cartilage repair (n = 5). (B) Semi-quantitative analysis of TB staining (n = 5). (C) Semi-quantitative analysis of COL-2 IHC (n = 5). (**P < 0.05* vs. Blank group, #*P < 0.05* vs. MSCs only group, ★*P < 0.05* vs. PEG-CHI group, ☆*P < 0.05* vs. PEG-CHI-KGN group)

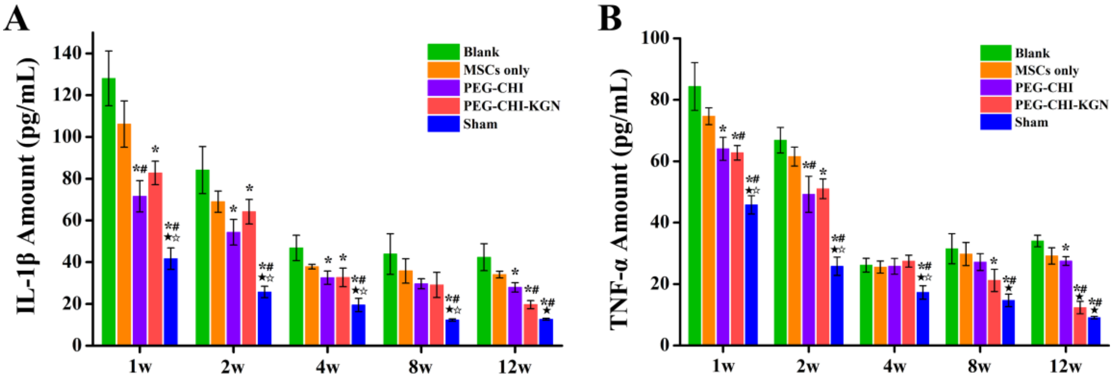


**Figure S10.** Synovial fluid analysis, levels of (A) interleukin 1β (IL-1β) and (B) tumor necrosis factor α (TNF-α) (n = 3). (**P < 0.05* vs. Blank group, #*P < 0.05* vs. MSCs only group, ★*P < 0.05* vs. PEG-CHI group, ☆*P < 0.05* vs. PEG-CHI-KGN group)

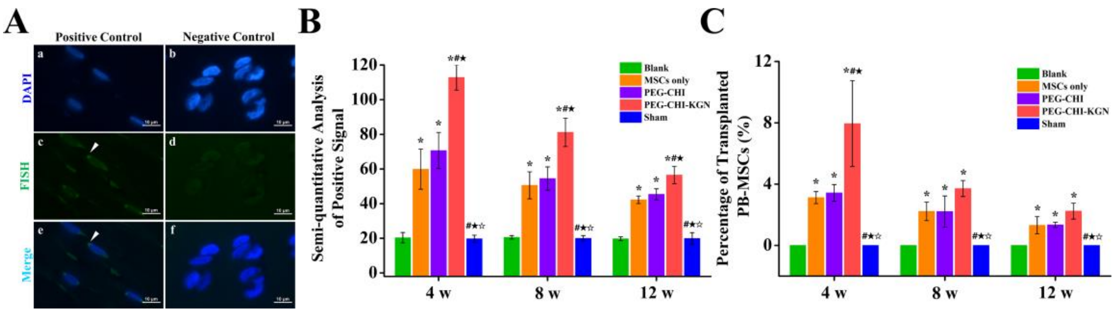


**Figure S11.** (A) Fluorescent in situ hybridization (FISH). (a, c, e) FISH was performed on normal articular cartilage from the trochlea of adult male rabbits (Positive signals were detected within chondrocytes. Positive Control);. (b, d, f) FISH was performed on normal articular cartilage from the trochlea of adult female rabbits (No signals are detected within chondrocytes. Negative Control) (Blue: DAPI, Green: FISH). (B) Semi-quantitative analysis of fluorescence intensity of positive signals (n = 3) (C) The proportion of transplanted PB-MSCs in regenerated cartilage after surgery for 4 weeks, 8 weeks and 12 weeks (n = 3). (*$P < 0.05$ vs. Blank group, $^{\#}P < 0.05$ vs. MSCs only group, ★$P < 0.05$ vs. PEG-CHI group, ☆$P < 0.05$ vs. PEG-CHI-KGN group).

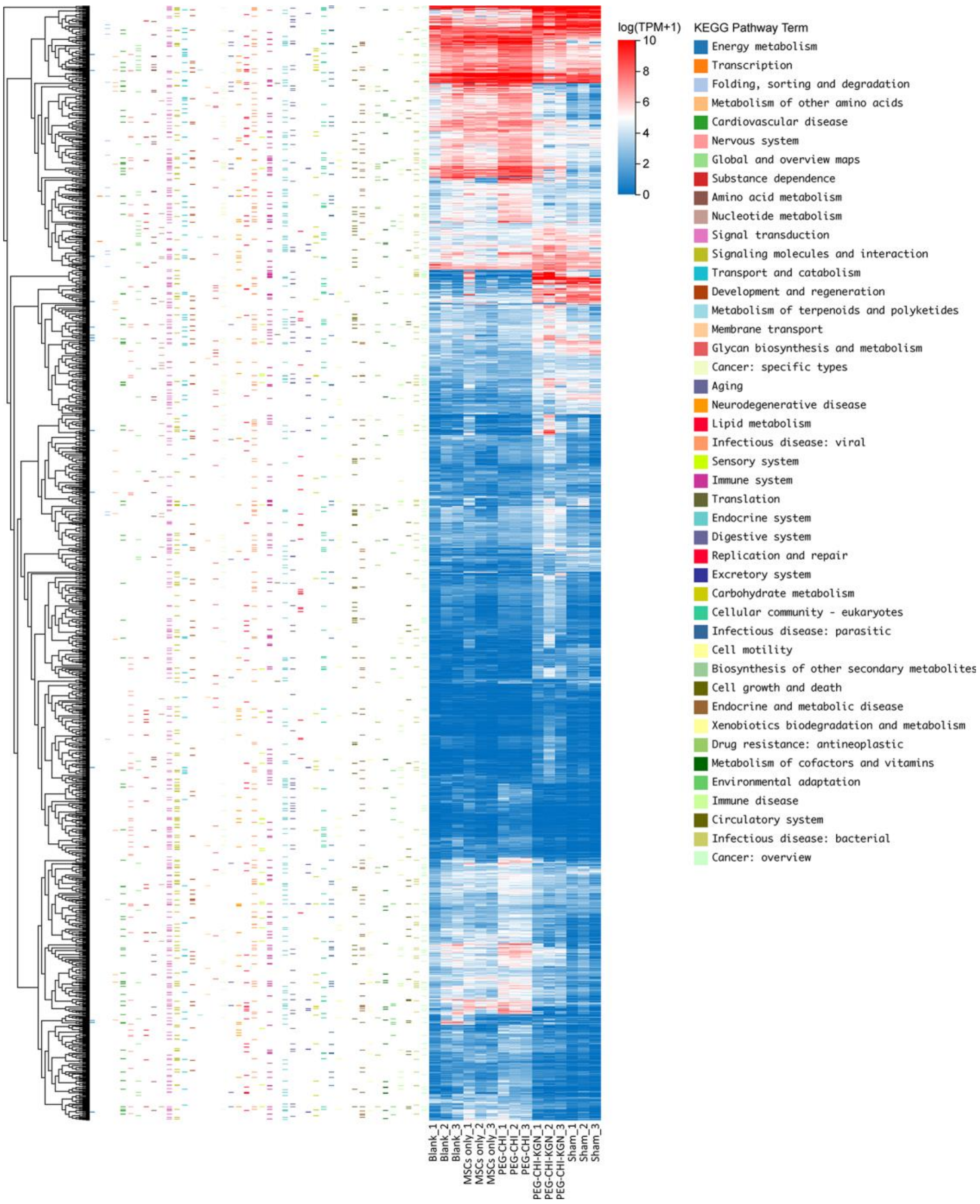


**Figure S12.** Heatmap and hierarchical clustering analysis of differentially expressed genes among the three groups assessed by RNA sequencing. Each column represents the expression profile of a tissue sample and each row corresponds to mRNA for log fold change ≥ 2 (n = 3, $*P < 0.05$).

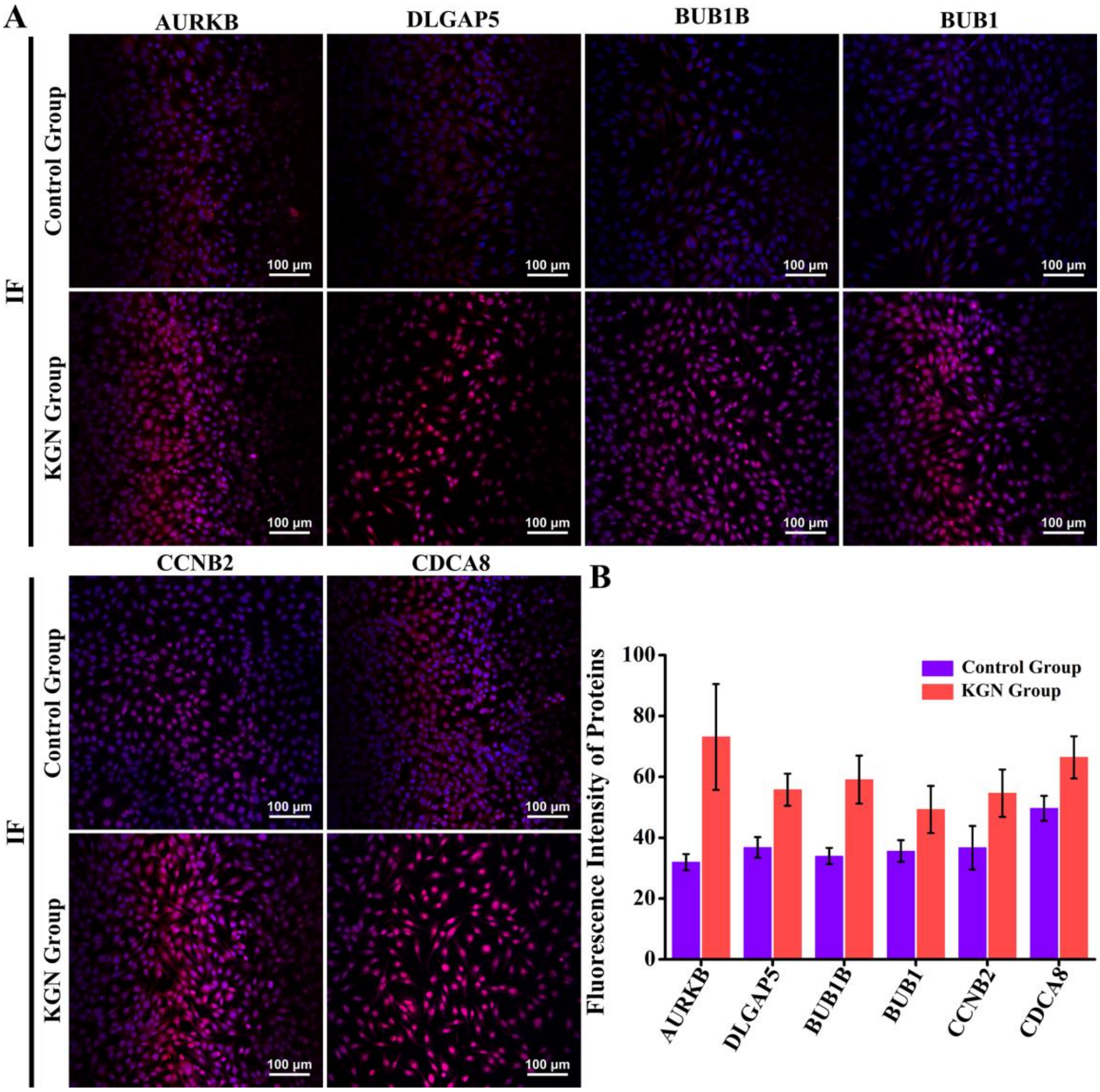


**Figure S13.** (A) The protein expressions of AURKB, DLGAP5, BUB1B, BUB1, CCNB2 and CDCA8 were detected by immunofluorescence staining (IF) after PB-MSCs were cultured in a medium with or without KGN for 2 weeks. (B) Semi-quantitative analysis of protein expression fluorescence intensity (n = 3, $*P < 0.05$).

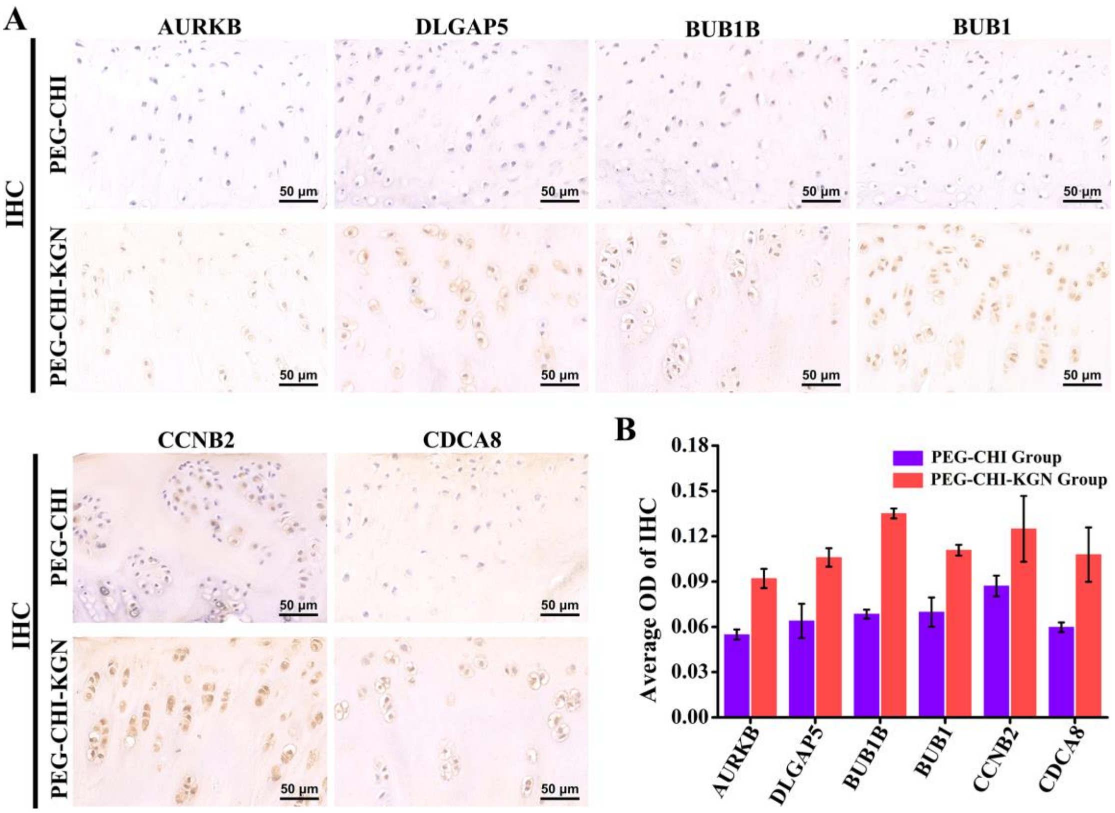


**Figure S14.** (A) Immunohistochemical staining (IHC) to detect the protein expression of AURKB, DLGAP5, BUB1B, BUB1, CCNB2 and CDCA8 in regenerated cartilage at 12 weeks in the PEG-CHI group and the PEG-CHI-KGN group (n = 3). (B) Semi-quantitative analysis of IHC results (n = 3, $^*P < 0.05$).

## References

[1] X. Li, J. Ding, Z. Zhang, M. Yang, J. Yu, J. Wang, F. Chang, X. Chen, *ACS Appl Mater Interfaces* **2016**, *8* (8), 5148, https://doi.org/10.1021/acsami.5b12212.

[2] W. L. Fu, C. Y. Zhou, J. K. Yu, *Am J Sports Med* **2014**, *42* (3), 592, https://doi.org/10.1177/0363546513512778.

[3] Y. Bu, H. Shen, F. Yang, Y. Yang, X. Wang, D. Wu, *ACS Appl Mater Interfaces* **2017**, *9* (3), 2205, https://doi.org/10.1021/acsami.6b15364.

[4] Z. Z. Zhang, D. Jiang, S. J. Wang, Y. S. Qi, J. Y. Zhang, J. K. Yu, *ACS Appl Mater Interfaces* **2015**, *7* (28), 15294, https://doi.org/10.1021/acsami.5b03129.

[5] Z. Z. Zhang, Y. R. Chen, S. J. Wang, F. Zhao, X. G. Wang, F. Yang, J. J. Shi, Z. G. Ge, W. Y. Ding, Y. C. Yang, T. Q. Zou, J. Y. Zhang, J. K. Yu, D. Jiang, *Sci Transl Med* **2019**, *11* (487), https://doi.org/10.1126/scitranslmed.aao0750.

[6] S. J. Wang, D. Jiang, Z. Z. Zhang, A. B. Huang, Y. S. Qi, H. J. Wang, J. Y. Zhang, J. K. Yu, *Sci Rep* **2016**, *6*, 36400, https://doi.org/10.1038/srep36400.
[7] G. Abedi, A. Sotoudeh, M. Soleymani, A. Shafiee, P. Mortazavi, M. R. Aflatoonian, *J Biomater Sci Polym Ed* **2011**, *22* (18), 2445, https://doi.org/10.1163/092050610X540503.
[8] Y. R. Chen, Z. X. Zhou, J. Y. Zhang, F. Z. Yuan, B. B. Xu, J. Guan, C. Han, D. Jiang, Y. Y. Yang, J. K. Yu, *Front Chem* **2019**, *7*, 745, https://doi.org/10.3389/fchem.2019.00745.
[9] M. P. van den Borne, N. J. Raijmakers, J. Vanlauwe, J. Victor, S. N. de Jong, J. Bellemans, D. B. Saris, S. International Cartilage Repair, *Osteoarthritis Cartilage* **2007**, *15* (12), 1397, https://doi.org/10.1016/j.joca.2007.05.005.
[10] A. Kawaguchi, H. Nakaya, T. Okabe, K. Tensho, M. Nawata, Y. Eguchi, Y. Imai, K. Takaoka, S. Wakitani, *Acta Orthop* **2009**, *80* (5), 606, https://doi.org/10.3109/17453670903350115.
[11] J. P. Meekel, G. Mattei, V. S. Costache, R. Balm, J. D. Blankensteijn, K. K. Yeung, *Acta Biomater* **2019**, *96*, 345, https://doi.org/10.1016/j.actbio.2019.07.019.
[12] Z. Li, H. Liu, Q. Zhong, J. Wu, Z. Tang, *FEBS Open Bio* **2018**, *8* (11), 1855, https://doi.org/10.1002/2211-5463.12533.
[13] J. Ye, J. Zha, Y. Shi, Y. Li, D. Yuan, Q. Chen, F. Lin, Z. Fang, Y. Yu, Y. Dai, B. Xu, *Clin Epigenetics* **2019**, *11* (1), 137, https://doi.org/10.1186/s13148-019-0723-0.
